# Ascaroside#18 Promotes Plant Defence by Repressing Auxin Signalling

**DOI:** 10.1111/ppl.70386

**Published:** 2025-07-11

**Authors:** Sharon Letia, Sabarna Bhattacharyya, Badou Mendy, Ute C. Vothknecht, Stephan H. von Reuss, Masaki Inada, Florian M. W. Grundler, M. Shamim Hasan

**Affiliations:** ^1^ INRES, Molecular Phytomedicine University of Bonn Bonn Germany; ^2^ Plant Cell Biology Institute for Cellular and Molecular Botany, University of Bonn Bonn Germany; ^3^ Laboratory for Bioanalytical Chemistry Institute of Chemistry, University of Neuchâtel Neuchâtel Switzerland; ^4^ Department of Biotechnology and Life Science Tokyo University of Agriculture and Technology Tokyo Japan

**Keywords:** ascr#18, auxin signalling, plant resistance, plant‐parasitic nematodes, susceptibility factors

## Abstract

Plant immunity against pathogens is primarily triggered by the perception of pathogen‐associated molecular patterns (PAMPs). Ascaroside#18, a nematode‐derived pheromone, is the first identified nematode‐associated molecular pattern conferring broad‐spectrum pathogen resistance. Recently, ascr#18 was shown to be recognised by the leucine‐rich repeat receptor *NILR1*, linked to pattern‐triggered immunity (PTI) against nematodes. However, the molecular mechanisms downstream of ascr#18 perception remain largely unknown. Here, we show that ascr#18 triggers an immune response that differs from the typical PTI features, with no reactive oxygen species burst or defence‐related growth inhibition. Further analysis indicates that the ascr#18‐associated resistance mechanism against cyst nematodes (CN) operates independently of the peroxisomal β‐oxidation pathway. Transcriptome profiling of Arabidopsis roots treated with ascr#18 revealed strong effects on the regulation of auxin transport and signalling genes, while classical defence genes remained unchanged. These changes, particularly the downregulation of auxin‐related genes, occur independently of *NILR1*. Analysis of CN feeding sites revealed that ascr#18 pretreatment reduced expression of the auxin influx carrier *AUX1* and the auxin‐responsive genes *SAUR69* and *IAA27*. Promoter‐reporter analysis confirmed reduced *AUX1* expression in both nematode‐infected and non‐infected roots treated with ascr#18. Since nematode establishment and the associated feeding cell development are heavily dependent on the modulation of auxin signalling, our results suggest a novel defence mechanism based on its suppression. This mechanism reduces nematode susceptibility without activating classical PTI responses. Our results provide new insights into how plants fend off biotrophic pathogens and point to ways of developing novel strategies for controlling nematodes and other biotrophic pathogens.

## 1 Introduction

Ascarosides (ascr) are small‐molecule pheromones that regulate development, reproduction and species interactions in animals (reviewed by Park et al. 2019). These signalling molecules were first identified in the model organism *Caenorhabditis elegans* as the ‘dauer pheromone’ (daumone#1, ascr#1, asc‐C7), which triggers larval diapause (Jeong et al. 2005). Subsequent research revealed that ascarosides influence numerous biological and social aspects of nematode behaviour beyond dauer formation. The basic ascaroside structure comprises a core l‐3,6‐dideoxy‐sugar (ascarylose) moiety linked to a (ω‐1)‐linked short‐chain fatty acid‐derived side chain of varying lengths (Jeong et al. 2005; Von Reuss et al. 2012). For example, ascr#2 (asc‐C6.MK) contains a 6‐carbon methylketone side chain, while ascr#3 (asc‐ΔC9) has an unsaturated 9‐carbon chain (Butcher et al. 2007). Structural diversity also arises from the attachment of additional building blocks from various primary metabolic pathways (Artyukhin et al. 2013; Butcher et al. 2009; Srinivasan et al. 2012).

Ascarosides represent evolutionarily conserved signalling molecules found throughout the Nematoda phylum, including free‐living *Caenorhabditis* species, entomopathogenic nematodes (Choe et al. 2012; Dong et al. 2018; Noguez et al. 2012), and animal‐ and plant‐parasitic genera (Manosalva et al. 2015; Shinoda et al. 2022).

The interactions between plant‐parasitic nematode (PPNs) and plants further revealed the diverse signalling functions of ascarosides. Manosalva et al. (2015) first reported the presence of ascr#18 (asc‐C11) (ascr#18) in PPNs, describing it as the most abundant and widely conserved across genera like *Meloidogyne*, *Heterodera* and *Pratylenchus*. Interaction studies demonstrated ascr#18's potency in eliciting immune responses against bacterial, fungal, viral and even other PPN species in both monocots and dicots (Klessig et al. 2019). The induction of salicylic acid (SA), jasmonic acid (JA) and related defence‐signalling pathways was identified as a key resistance mechanism, establishing ascr#18 as the first nematode‐associated molecular pattern (NAMP) to be characterised (Manosalva et al. 2015). The understanding of ascaroside–plant interactions took an unexpected turn when Manohar et al. (2020) reported that plants can metabolically edit ascr#18 (asc‐C11) into a shorter‐chain variant, ascr#9 (asc‐C5), through the peroxisomal β‐oxidation pathway. This metabolic editing produces ascr#9, which acts as a deterrent, repelling nematodes from the plant. Metabolomic analysis of [^13^C_2_]‐labelled ascr#18‐treated tomato plants confirmed the production of [^13^C_2_]‐ascr#9, validating this metabolic conversion. Additionally, ascr#9 (asc‐C5) has also been reported to be a potent elicitor of plant immunity through activation of defence‐marker genes (Manohar et al. 2020).

So far, ascr#18 remained an ‘orphan’ NAMP, with its cognate pattern recognition receptor (PRR) eluding identification. Recently, however, a novel study has reported that ascr#18 is perceived by a leucine‐rich repeat (LRR) membrane‐localised protein, called nematode‐induced leucine‐rich repeat 1 (*NILR1*) (Huang et al. 2023). *NILR1* was found to be upregulated in Arabidopsis upon treatment with water containing surface molecules of infective juveniles of *H. schachtii* (NemaWater) and was shown to be able to activate plant immune responses. The effect of NemaWater was strongly reduced after heat and proteinase K treatment, as shown by reduced plant immune responses (Mendy et al. 2017). This has led to the conclusion that *NILR1* may recognise proteinaceous ligands. Nonetheless, evidence has been provided for the physical interaction between ascr#18 and the ligand‐binding domain of NILR1 in both Arabidopsis and potato (Huang et al. 2023; Huang, Yuan, Ramirez, Zhao, et al. 2024). They also demonstrate that the *nilr1* mutant is insensitive to ascr#18, failing to exhibit heightened immunity against nematodes and bacteria observed in Col‐0 Arabidopsis treated with ascr#18. While the identification of *NILR1* as the receptor for ascr#18 sheds light on the recognition of nematode signals and subsequent defence responses, understanding the broader network of involved elements of immunity, particularly phytohormone signalling, remains crucial for understanding plant–nematode interactions.

Phytohormones play a complex, dual role in plant–nematode interactions, acting as both positive regulators of host defence and targets of manipulation by PPNs to aid parasitism (Sikder et al. 2021). As positive regulators, SA (Branch et al. 2004) and JA (Bhattarai et al. 2008) have been linked to enhanced host resistance against a broad spectrum of PPNs across different plant species. Exogenous application of SA and JA has been reported to confer increased resistance to various *Meloidogyne* species, like *M. incognita* , *M. javanica* and *M. chitwoodi* (Fujimoto et al. 2011; Molinari 2016; Vieira dos Santos et al. 2013), as well as *Heterodera* species (Kammerhofer et al. 2015). Hormonal crosstalk also plays a significant role in PPN resistance, as exemplified by the synergistic action of JA and the gaseous phytohormone ethylene in eliciting systemic defence responses in rice, conferring resistance to *M. graminicola* (Nahar et al. 2011). On the other hand, auxin is probably the phytohormone most often manipulated by PPNs to support their parasitism. PPNs are able to hijack auxin biosynthesis, auxin transport and auxin signalling, a pathway that is crucial for plant growth and cell expansion, but also for the formation and maintenance of their food sources, such as syncytia and giant cells (reviewed by Gutierrez et al. 2009; Oosterbeek et al. 2021). Kyndt et al. (2016) showed that *M. incognita* manipulates the auxin influx and efflux genes, *AUX1* and *PINs*, respectively, to facilitate feeding site establishment. Specifically, *M. incognita* upregulates the influx‐related *AUX1* in the early stages of giant cell formation to allow auxin accumulation, while downregulating the efflux‐related PIN in galls to prevent auxin leakage. Simultaneously, *Meloidogyne* upregulates PIN4 to stabilise hormone levels (Kyndt et al. 2016). Similarly, Arabidopsis auxin loss‐of‐function mutants, such as *axr2/iaa7*, showed decreased susceptibility to *H. schachtii* infection, with smaller and deformed feeding sites compared to wild‐type (Goverse et al. 2000). Furthermore, chemical inhibition of auxin transport using N‐(1‐naphthyl) phthalamic acid (NPA) also led to reduced susceptibility to *H. schachtii* , confirming that auxin aids PPN parasitism in hosts (Goverse et al. 2000). This phenomenon extends beyond plant–nematode interactions. An intact auxin signalling pathway has been reported as a prerequisite for normal host invasion and establishment by the bacterial pathogen *Pseudomonas syringae* . Impaired auxin responses in *axr2* loss‐of‐function mutants also led to reduced susceptibility to *P. syringae* (Wang et al. 2007; Djami‐Tchatchou et al. 2020).

While ascarosides are well established as key signalling molecules in nematode development, behaviour and interspecies interactions, their broader roles in plant–nematode dynamics remain poorly understood. Specifically, although ascr#18 has been identified as a NAMP that activates plant immune responses, the precise mechanisms underlying its perception and downstream signalling are still unclear. In this study, we reveal that ascr#18 triggers a non‐canonical immune response without the involvement of *NILR1*, suppressing auxin transport and signalling. This suppression significantly enhances Arabidopsis resistance to cyst nematodes and potentially confers broad‐spectrum protection against a wide range of PPNs and other plant pathogens.

## 2 Materials and Methods

### 2.1 Plant Growth and Nematode Infection

*A. thaliana* ecotype Col‐0 and *acx1acx5* (Manohar et al. 2020) seeds were sterilised using 0.6% sodium hypochlorite and 70% ethanol under a clean bench. The seeds were then rinsed three times with sterile tap water prior to air drying for 1–2 h. Seeding was done on agar medium supplemented with KNOP nutrient solution for 12 days prior to inoculation. The growing conditions were as follows: plates were placed in a room maintained at approximately 23°C under a 16/8‐h long day/dark regimen (Anwer et al. 2018). To hatch *H. schachtii* infective juveniles, 300 sterile cysts were suspended in 3 mM zinc chloride solution within a sterile hatching chamber. The chamber was incubated in complete darkness for at least 5 days to induce hatching. The emerged second‐stage juveniles (J2s) were collected and washed with sterile tap water to remove residual zinc chloride before inoculation. Plant inoculation was performed following the protocol described in Hasan et al. (2022). Briefly, 12‐day‐old seedlings were inoculated with 60–70 J2s per plant. Each genotype/treatment included a minimum of 15 plants per biological replicate. Male and female nematodes were counted using a Leica stereomicroscope at 12 days post inoculation (dpi), while female and female‐associated syncytia sizes were measured at 14 dpi. Images of females and female‐associated syncytia were captured with the M165C stereomicroscope, and quantitative size parameters were obtained using the LAS v. 4.3 image analysis software from Leica Microsystems.

### 2.2 Growth Inhibition Assays

*A. thaliana* (ecotype: Col‐0) seeds were sown on half‐strength Murashige and Skoog (MS) media and grown for 5 days. At 5 days post‐seeding (dps), the seedlings were transplanted into 6‐well plates containing liquid MS media. The media was supplemented with the respective ascr#18 concentrations, flg22 as a positive control, and an equivalent amount of sterile tap water as a negative control (mock). At 12 dps, data were collected to quantify plant growth parameters, including fresh weight, root length and root surface area. Fresh weight was measured using a precision laboratory balance. Root images for phenotyping root length and root surface area were obtained using the Epson root scanners, and measurements were done using the WinRhizo application.

### 2.3 ROS Analyses

To analyse the ROS profile in leaves upon ascr#18 treatment, 4 mm‐wide leaf discs were excised using a cork borer from 4‐week‐old Arabidopsis plants. The luminol‐based ROS assay was performed as described in previous work (Mendy et al. 2017). Briefly, the leaf discs were suspended in water overnight, then transferred to 96‐well plates and allowed to rest for 1 h prior to subjecting them to reagents. To each leaf disc, the following reagents were added: 15 μL of 20 μg mL^−1^ horseradish peroxidase, 35 μL of 10 mM L‐012 sodium salt (8‐amino‐5‐chloro‐2,3‐dihydro‐7‐phenyl‐pyrido[3,4‐d]pyridazine‐1,4‐dione, sodium salt) (L‐012, Wako Chemicals), and 50 μL of 0.001, 0.01 and 1 μM ascr#18 (asc‐C11). Equal amounts of 1 μM flg22 (GenScript) and water were used as a positive and negative control, respectively. ROS production, indicated by light (RLU) emissions, was measured by the luminometer over short (1.5 h), medium (3.5 h) and long (6 h) time periods. For every biological replicate, four technical replicates were used per treatment, and the experiments were repeated on at least three independent occasions.

### 2.4 Nematode Infection Site Collection

Twelve‐day‐old Arabidopsis Col‐0 plants were grown on KNOP media and treated with either 1 mL/plant of 1 μL of ascr#18 or sterile autoclaved tap water as a mock. Plants were categorised into three experimental groups: Mock‐ *H. schachtii* ‐uninfected (Mock/Ui), Mock‐*H. schachtii*‐infected (Mock/i) and ascr#18‐ *H. schachtii* ‐infected (ascr#18/i). Six hours after ascr#18 or mock treatment, the ‘infected’ groups were inoculated with 60–80 *H. schachtii* J2s per plant. At 3 days post‐infection (dpi), the infection sites, approximately 2 mm from the ‘infected’ category, were excised with the aid of sharp blades and fine tweezers under the LEICA stereo microscope (Siddique et al. 2014). Uninfected‐water‐treated Col‐0 roots were also cut as controls (Mock/Ui). For all samples, root tips and lateral roots were deliberately excluded during tissue collection. All samples were collected directly into liquid nitrogen and stored at −80°C until further processing for RNA extraction, cDNA synthesis and finally gene expression analysis.

### 2.5 Gene Expression Analysis (RT‐qPCR)

Total RNA was extracted from harvested root samples using the Quick‐RNA Miniprep kit (Zymo Research) according to the manufacturer's protocol. Genomic DNA contamination in RNA samples was eliminated using the DNA‐free DNA Removal Kit (Ambion). Reverse transcription of RNA into cDNA was done using the High Capacity cDNA Reverse Transcription Kit (Life‐Technologies, cat. no. 4368814) according to the manufacturer's instructions. Transcript abundance in the samples was measured using the StepOnePlus Real‐Time PCR System (Applied Biosynthesis). Each 20‐μL PCR reaction contained: 10 μL Fast SYBR Green qPCR Master Mix, 0.5 μL each of forward and reverse primers, 1 μL of cDNA template and 8 μL of nuclease‐free water. The 18S rRNA and UBQ10 genes were used as endogenous controls as specified in the figure legends. For the amplification of the 18S ribosomal RNA gene, cDNA templates were diluted at a ratio of 1:100 prior to use in the PCR in order to optimise the reaction conditions and prevent potential saturation effects due to the high abundance of 18S transcripts. Relative expression of the genes of interest was determined by normalising their CT values to those of the endogenous controls. Each biological replicate represents the mean of three technical replicates, and the final data represent the average of three biological replicates. All gene expression values were normalised to the mean expression levels of target genes in mock‐treated Col‐0 roots from the first biological replicate, which served as the reference sample (Guarneri et al. 2023). Differences between means were evaluated by one‐way ANOVA, followed by Tukey's post hoc test. Statistical significance (*p* < 0.05) is indicated by different letters above the bars. Primer sequences used are indicated in the Supporting Information (Table S1).

### 2.6 RNA Sequencing and Data Analyses

RNA sequencing was performed to investigate the various changes in transcriptional landscape when plants were treated with ascr#18. Briefly, plants were grown on ½‐strength KNOP medium, and 12‐day‐old seedlings were transferred to liquid KNOP medium in 6‐well plates supplemented with either 1 μM ascr#18 or sterile water as a mock treatment. Root samples were collected after a 6‐h treatment period. RNA Sequencing was carried out with four biological replicates. 3′ mRNA Sequencing was carried out at the NGS Core Facility Service of the University Hospital Bonn, on a NovaSeq6000 (Illumina) platform. cDNA libraries were synthesised using the QuantSeq protocol using standard oligo dT primers without any removal of the ribosomal RNA (Moll et al. 2014). The obtained fastq files were then analysed using fastQC to check for quality score, GC content, etc. Universal adapter removal was carried out using cutAdapt (Martin 2011). Alignment with the paired‐end reads was done against the *Arabidopsis thaliana* reference genome (TAIRv10) using HISAT2 (Kim et al. 2019) with a HISAT index. The detailed read and mapping statistics have been added as Table S2. Gene counts from the aligned files were produced using the featureCounts program (Liao et al. 2014) from the Rsubread (Liao et al. 2019) package using default parameters. Differential expression analyses were performed using DESeq2 after creation of a contrast matrix (ascr#18 vs. mock). The criteria for DEG (differentially expressed gene; Table S3) in our study were considered to be FDR < 0.05, based on the false discovery rate method. GO (gene ontology) analyses of the obtained DEGs were performed using shinyGO (Ge et al. 2020).

### 2.7 Analysis of pro*AUX1* :YFP‐ *AUX1* Upon Ascr#18 Treatment and Nematode Infection

For microscopy analyses, the previously established constitutive pro*AUX1*:YFP‐*AUX1* transgenic line in WT background (Col‐0) (Swarup et al. 2004) was used. YFP fluorescence was perceived through a Leica SP8 Lightning Confocal Microscope (Leica Microsystems) at a 10× magnification. For excitation, an argon laser with a wavelength of 514 nm was used, and emission was detected using a Hyd detector at the range of 516–546 nm.

### 2.8 GUS Staining

GUS promoter‐reporter analysis was carried out following the protocol described by Chopra et al. (2021). The DR5::GUS (Stepanova et al. 2008) stable reporter lines were grown on Knop media, and at 12 days old, the plants were treated either with water (Mock) or 1 μM ascr#18. Following these treatments, the plants were infected with the CN *H. schachtii* . The roots of the various samples were incubated with X‐gluc at 37°C for over 8 h. The reaction was then stopped, and the samples were thoroughly washed with 70% ethanol. Staining was performed 3 days post‐infection (3 dpi). The stained syncytia were imaged using a KEYENCE BZ‐X810 microscope.

### 2.9 Statistical Analysis

The GraphPad Prism software (version 10.4.0) for Windows was used for statistical analysis and graphical illustrations. For infection assays, each biological replicate included 20–25 plants per genotype or treatment, and experiments were independently repeated at least 3 separate occasions. The assumption of normal distribution was tested using Shapiro–Wilk and Kolmogorov–Smirnov analyses (*p* > 0.05). The specific statistical methods used for each experiment are listed in the figure legends. Statistical significance between experimental groups is denoted by different lowercase letters above the bars or data points as an outcome of ANOVA, followed by Tukey's post hoc HSD tests.

## 3 Results

### 3.1 Pretreatment of *A. thaliana* With Ascr#18 Confers Resistance to *H. schachtii*

**FIGURE 1:**
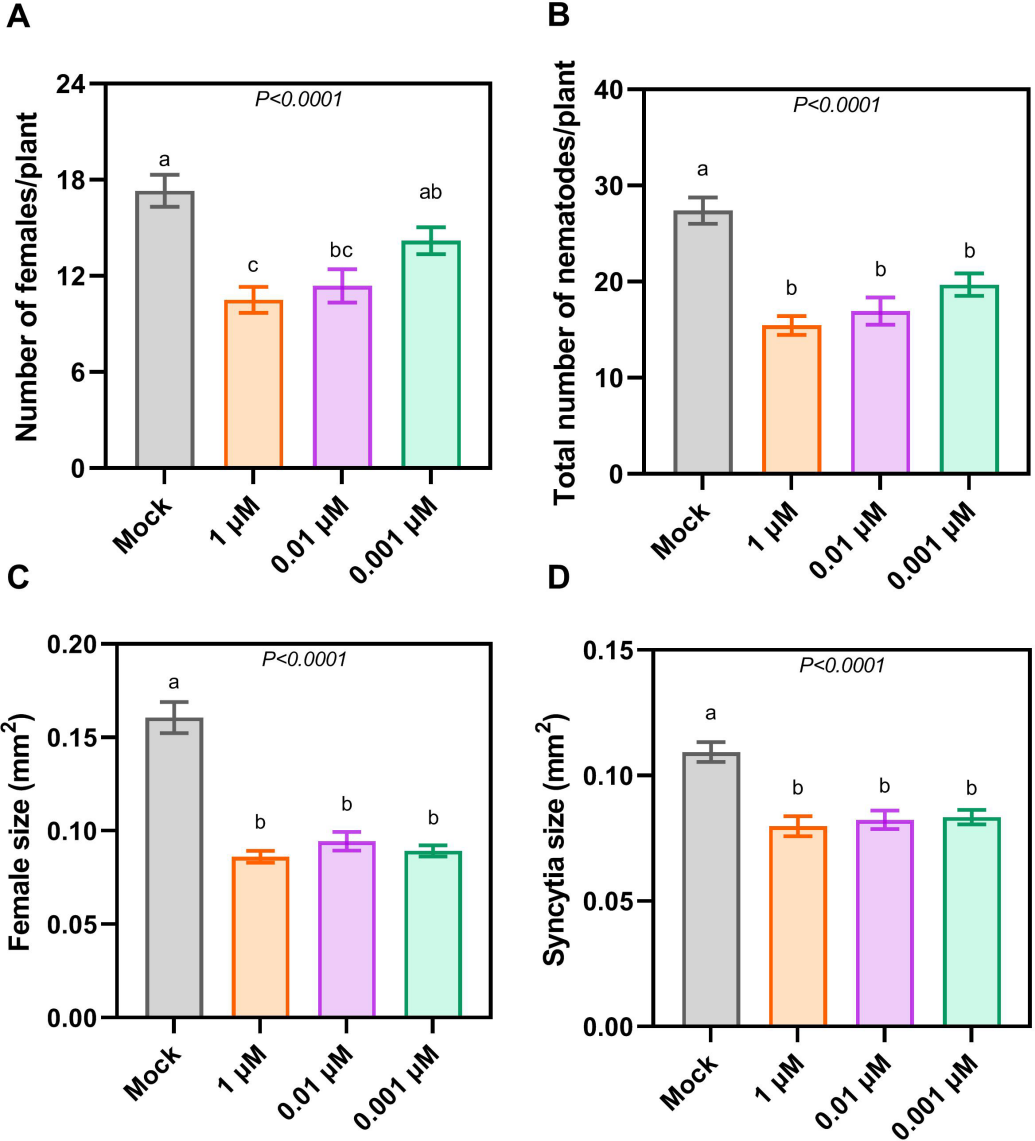
ascr#18 elicits immune responses in *A. thaliana* . Treatment of 12‐day‐old *A. thaliana* Col‐0 plants with different concentrations of ascr#18 for at least 24 h prior to *H. schachtii* inoculation conferred heightened resistance. (A, B) Plants pretreated with all three ascr#18 concentrations had significantly lower numbers of females and total nematode numbers (males and females) compared to those pretreated with sterile water as a negative control. Average numbers of females and total numbers of nematodes (males and females) respectively counted at 12 dpi using a Leica stereomicroscope. The experiment was repeated three independent times and each biological replicate had 20 plants each per treatment. (C, D) Ascaroside‐pretreated plants have significantly smaller female and female‐associated syncytia in comparison to their water‐treated counterparts. Average sizes of females and corresponding syncytia were measured at 14 dpi; a total of 30 independent female‐syncytia pairs were measured per biological replicate. Images were captured with the M165C stereomicroscope and quantitative size parameters obtained using the LAS v. 4.3 image analysis software from Leica Microsystems. Results represented are an average across three independent biological repetitions ± SE. Letters above the bars indicate significant differences across treatments (*p* < 0.05; ANOVA).

To confirm the potency of ascr#18 as an elicitor, 12‐day‐old Col‐0 Arabidopsis plants were pretreated with three concentrations (0.001, 0.01 and 1 μM) of ascr#18 or sterile water as a mock for 24 h before inoculation with *H. schachtii* infective stage juveniles (J2s). At 12 days post‐infection (dpi), we quantified the number of established nematodes, while the size of females alongside their corresponding syncytia was measured at 14 dpi. All tested ascr#18 concentrations induced immune responses in *A. thaliana* , evidenced by significant reductions in both the number of females (Figure 1A) and total nematode counts (Figure 1B) compared to mock‐treated controls. Notably, plants treated with ascr#18 also had significantly smaller females and female‐associated syncytia (Figure 1C,D) compared to their mock‐treated counterparts. These results demonstrate that ascr#18 elicits immune responses that limit *H. schachtii* root infection but also restrict the nematode's ability to establish and maintain viable feeding sites.

### 3.2 Ascr#18 Shows a Distinct Lack of Canonical Immune Response Activation in *A. thaliana*

**FIGURE 2:**
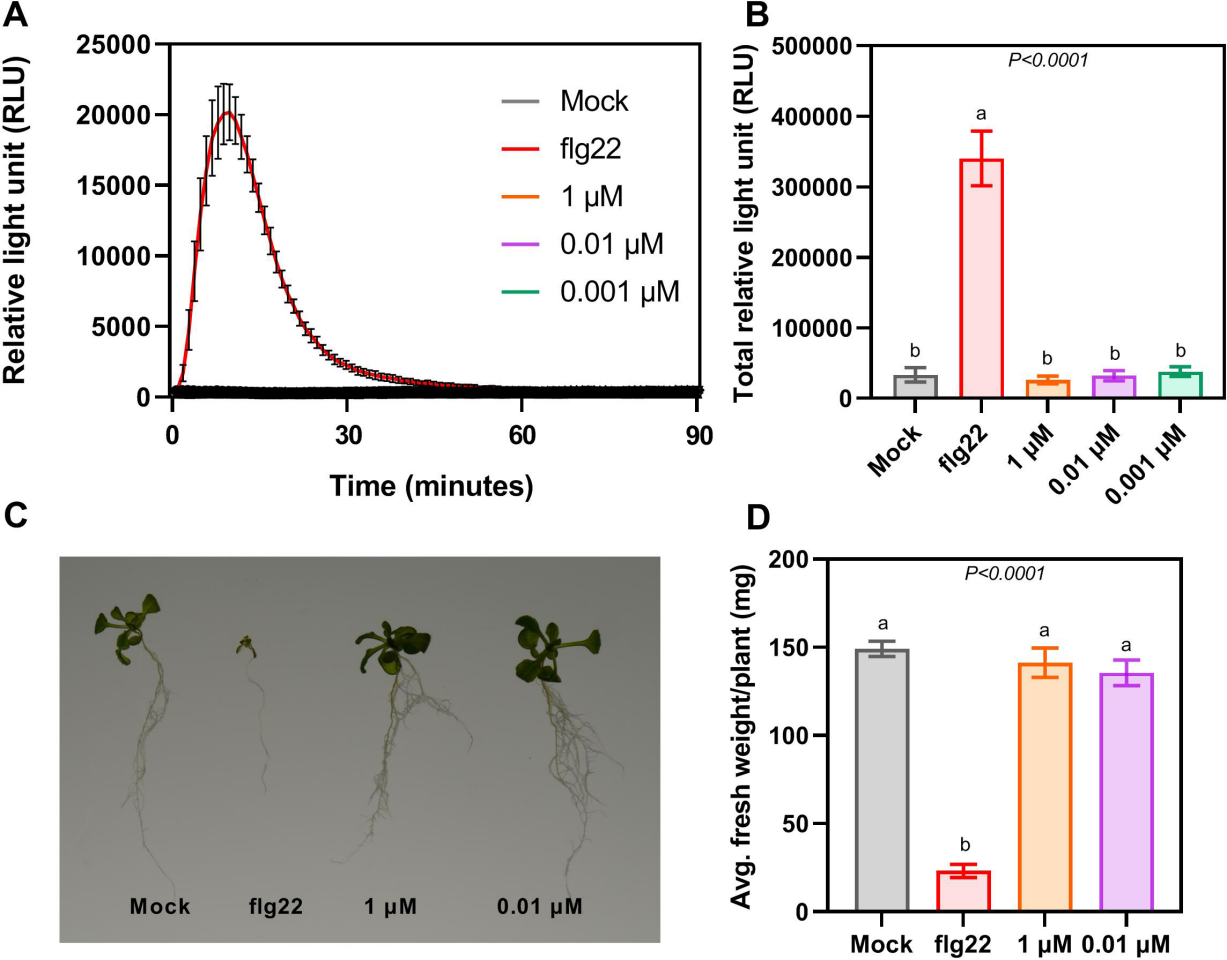
ascr#18 does not induce typical immune responses in *A. thaliana* . ROS bursts and defence‐associated growth inhibition are considered classical immune responses upon ligand perception; however, these were not observed upon ascr#18 perception. (A) Col‐0 leaf discs were cut and treated with water, different ascr#18 concentrations, and flg22, a bacterial peptide as a positive control. ROS bursts in the form of relative light units (RLU) were measured using the luminol‐based assay for 90 min. flg22 triggered typical defence‐related ROS bursts, but all three concentrations of ascr#18 had no significant ROS burst. The mean ± SE of three technical replicates was plotted per minute. The experiment was repeated three times independently, yielding the same results. (B) total amount of ROS produced over 90 min post‐elicitor addition. Total ROS accumulated in both ascarosides and water were comparable, while flg22 had significantly higher ROS accumulated. Letters above bars represent statistical differences between treatments (*p* < 0.05, ANOVA). (C, D) Five‐day‐old Col‐0 seedlings were transplanted into six‐well plates containing water, ascr#18 and flg22; fresh weight was measured at 12 dps, 7 days post‐transplanting. Fresh weight in flg22 was drastically impaired, whereas seedlings treated with ascr#18 did not exhibit any growth inhibition. (C) image of *A. thaliana* seedlings taken at 7 days post‐treatment (dpt). Images are representative of 12 plants of each treatment group. (D) average fresh weight measured at seven dpt. Each treatment had 12 plants per biological repetition. The experiment was conducted on three independent occasions. Bars represent means ± SE, *n* = 12, and letters show significant differences (*p* < 0.05, ANOVA).

Defence‐associated transcriptional changes in *A. thaliana* following ascr#18 treatment were previously reported by Manosalva et al. (2015). Their study showed upregulation of PTI‐marker genes involved in SA, JA and MAPK signalling cascades at different time points after ascr#18 treatment (Manosalva et al. 2015). However, other hallmarks of PTI remained unexplored. To address this gap, we investigated two canonical PTI responses: ROS burst and defence‐related growth inhibition (Hasan et al. 2024). Previously, Ning et al. (2020) reported the absence of ROS burst upon ascr#18; however, they only tested one ascr#18 concentration (1 μM) at one time period. Here, we explore different ascr#18 concentrations (0.001, 0.01 and 1 μM) at different time exposures. ROS bursts were measured in Col‐0 leaf discs treated with either water (mock), three concentrations of ascr#18, or flg22 (positive control) using a luminol‐based assay over multiple time intervals (short, medium and long). While flg22‐treated leaf disc samples exhibited the typical ROS burst curve, no such pattern was observed in ascr#18‐treated leaf discs (Figures 2A and S1). Total ROS accumulation over the length of the experiment, quantified as total RLU, was comparable between ascr#18 and mock (Figures 2B and S1). Next, we performed a growth inhibition assay by transferring 5‐day‐old Col‐0 seedlings to media supplemented with mock, three concentrations of ascr#18, or flg22. Unlike flg22 treatment, which caused typical defence‐associated growth reduction, ascr#18‐treated seedlings showed no significant differences in plant growth parameters, including fresh weight, compared to mock‐treated controls (Figures 2C,D and S2). These results suggest that ascr#18‐mediated resistance operates through a PTI‐independent mechanism distinct from the classical NAMP–PRR pathway characterised by rapid ROS bursts.

### 3.3 Alternative Ascr#18‐Associated Resistance Mechanism Beyond Metabolic Editing

In light of alternative ascr#18 resistance mechanisms, recent work by Manohar et al. (2020) demonstrated that *A. thaliana* metabolically converts ascr#18 (asc‐C11) to ascr#9 (asc‐C5) through the peroxisomal β‐oxidation pathway, with ascr#9 acting as a nematode repellent. Acyl‐CoA oxidases (ACX) function upstream of the β‐oxidation pathway, where they fix α,β‐unsaturation in the fatty acid side chain. Manohar et al. (2020) confirmed that the Arabidopsis double mutant void of acyl‐CoA oxidases 1 and 5 (*acx1acx5*) carries an impaired β‐oxidation pathway, evidenced by increased accumulation of ascr#18 (asc‐C11) and significantly lower amounts of ascr#9 (asc‐C5) in comparison to Col‐0, thus making this mutant a good genotype to characterise ascr#18‐induced defence in Arabidopsis.

**FIGURE 3:**
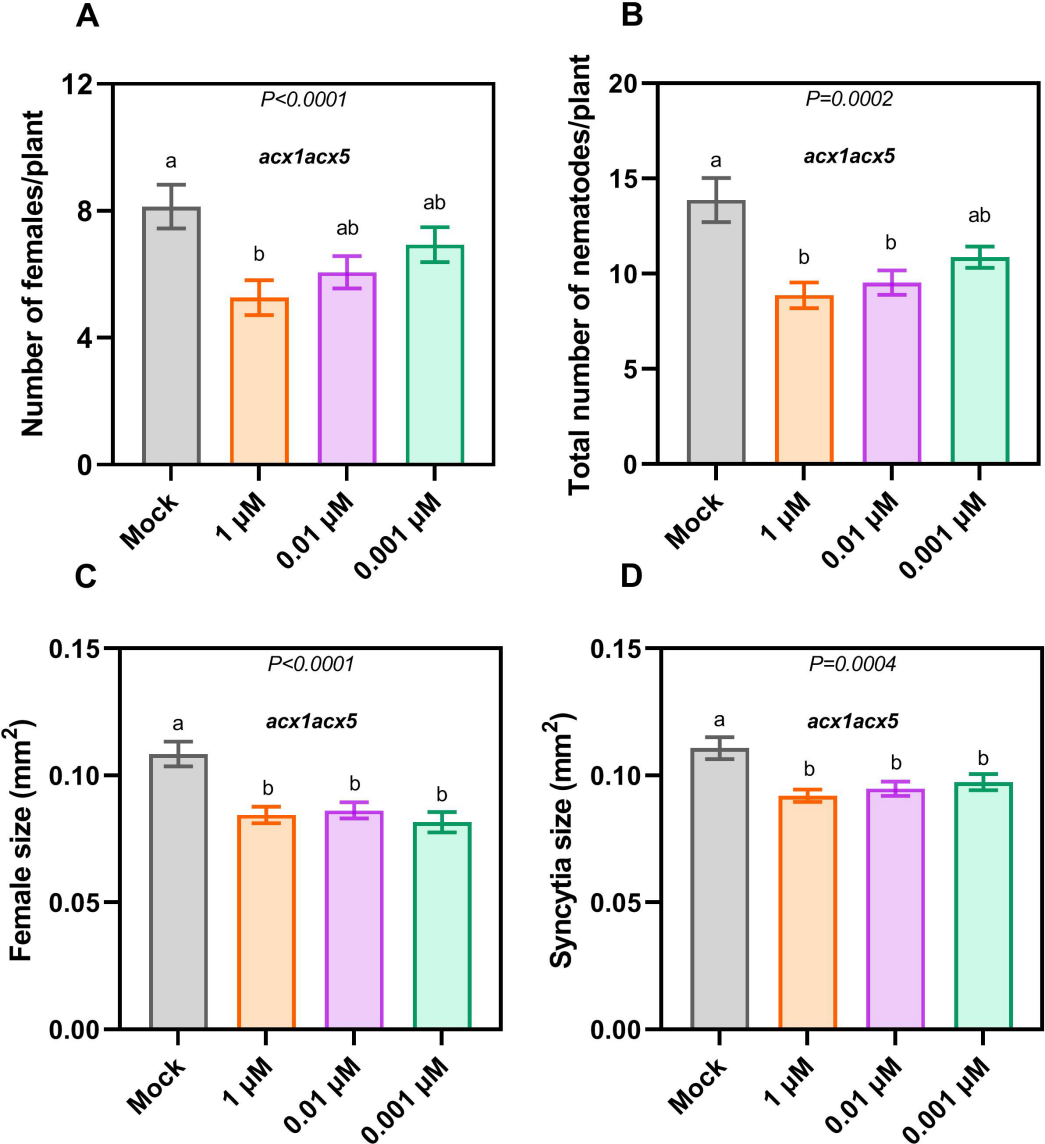
ascr#18‐associated resistance remains intact in the absence of its metabolic editing to ascr#9. Peroxisomal β‐oxidation breaks down ascr#18 to ascr#9, which then acts as a repellent against nematodes. β‐oxidation loss‐of‐function double mutants, *acx1acx5*, maintained heightened resistance to *H. schachtii* post ascr#18 pretreatment. (A, B) 12‐day‐old *acx1acx5* were pretreated with three ascr#18 concentrations and sterile water as a control. All three concentrations of ascr#18 had significantly lower numbers of females and total nematode numbers (males and females) compared to those pretreated with sterile water as a negative control. The number of females and total number of nematodes (males and females) were counted at 12 dpi using a Leica stereomicroscope. (C, D) At 14 dpi, female sizes and corresponding syncytia were measured. All three ascr#18 concentrations (0.001, 0.01 and 1 μM of 3 mM ascr#18) induced the formation of smaller feeding sites (syncytia) and consequently smaller females in *acx1acx5* double mutants compared to the water‐treated mutants. Images were captured with the M165C stereomicroscope, and quantitative size parameters obtained using the LAS v. 4.3 image analysis software from Leica Microsystems. A total of 30 independent female‐syncytia pairs were measured per biological replicate. Results represented are an average across three independent biological repetitions ± SE. Letters above the bars indicate significant differences across treatments (*p* < 0.05; ANOVA).

To investigate whether ascr#18 retains its efficacy in inducing immune responses in the absence of the peroxisomal β‐oxidation pathway, we performed an infection assay using *H. schachtii* and *acx1acx5*. The double mutant was pretreated with ascr#18 or mock 24 h prior to inoculation. Nematodes were counted at 12 dpi, whereas female and syncytia sizes were analysed at 14 dpi. Despite the impaired ability to convert ascr#18 to ascr#9, ascr#18‐treated *acx1acx5* mutants showed enhanced resistance to *H. schachtii* as opposed to mock‐treated ones. The number of females (Figure 3A) and the total nematode counts (Figure 3B) in the ascr#18‐treated *acx1acx5* remained significantly lower than those in the mock‐treated *acx1acx5* plants, mirroring our observations in Col‐0 plants (Figure 1). Furthermore, the sizes of females (Figure 3C) and their corresponding syncytia (Figure 3D) were also significantly smaller in the ascr#18‐treated *acx1acx5* plants. Interestingly, *acx1acx5* plants show neither compromised resistance to *H. schachtii* nor any alteration in flg22‐induced ROS bursts compared to Col‐0 (Figures S3 and S4). Additionally, these plants fail to exhibit ascr#18‐induced ROS bursts (Figure S3). These results indicate that the metabolism of ascr#18 may not contribute to or at least is not sufficient to confer resistance to *H. schachtii* , and suggest that, beyond the metabolic editing of ascr#18, there may be an independent resistance mechanism that the plant employs to defend itself against PPNs upon ascr#18 perception.

### 3.4 Ascr#18 Treatment Suppresses Genes Associated With Auxin Response and Transport

**FIGURE 4:**
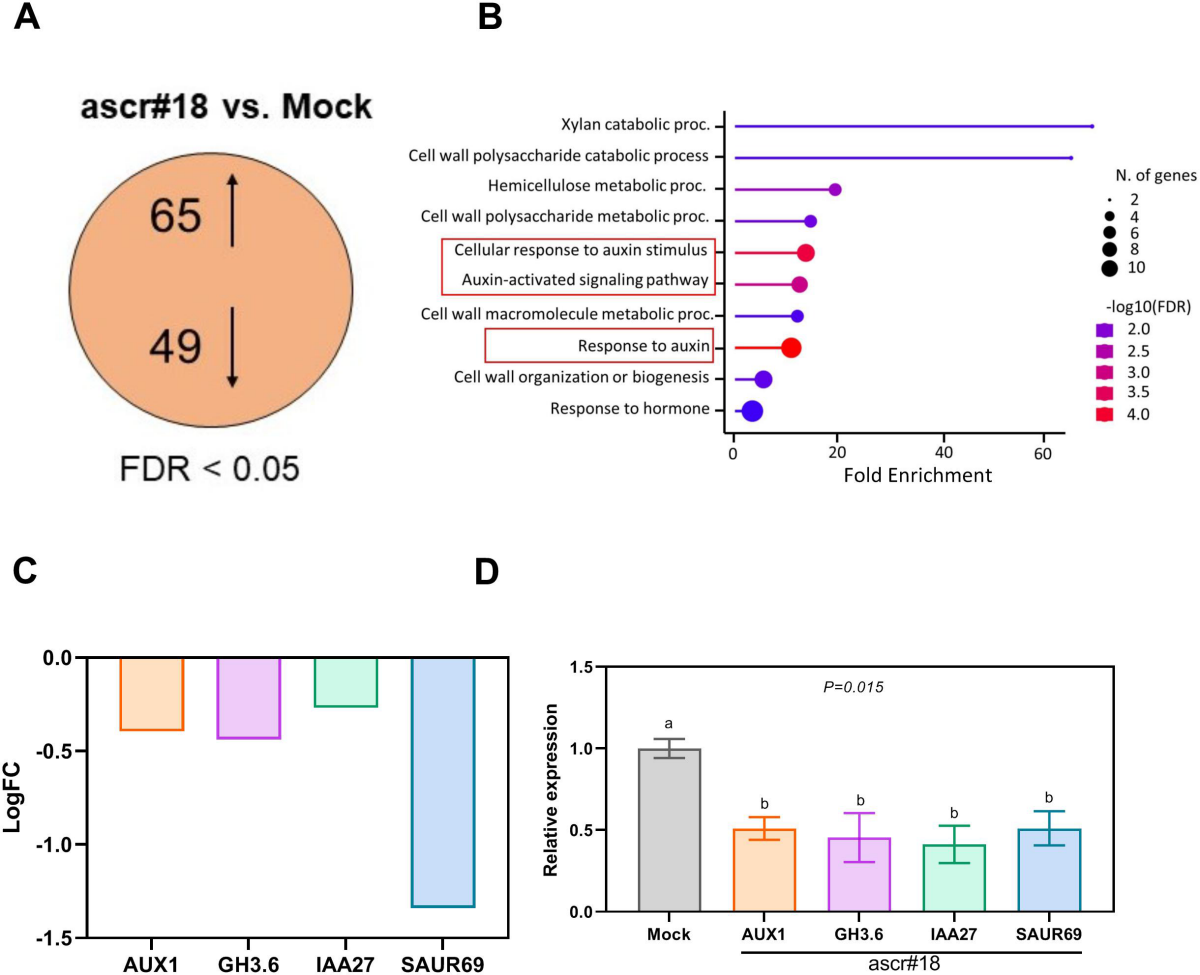
Differential expression analyses (FDR < 0.05) between ascr#18 treated and control (mock) samples. (A) Venn diagram representing the total number of obtained DEGs. (B) Top 10 GO (gene ontology) terms associated with biological processes obtained with the downregulated genes. The terms related to auxin have been highlighted in red. (C) log fold changes of the various auxin signalling genes (ascr#18 vs. mock). (D) RT‐qPCR confirmation of the genes shown in subpart (C). Bars represent means ± SE of three biological repeats.

In order to investigate the transcriptional reprogramming of plants upon treatment with ascr#18, we performed RNA‐Seq analyses of plants treated with Ascr#18. As evident from Figure 4A, at an FDR cutoff of 0.05, a total of 114 DEGs could be obtained (Table S3). However, most of the obtained DEGs had a log2FC much less than 1, which could be attributed to the trivial effect of ascr#18 on the transcriptomic landscape of Arabidopsis. There were a total of 65 upregulated genes, which mostly yielded stress‐responsive GO terms, with scattered and redundant genes not strictly belonging to any signalling pathway (Figure S5). Interestingly, only a single known PTI‐related gene, *PR4* (*PATHOGENESIS RELATED 4*) was upregulated in our dataset, appearing in two GO terms: positive regulation of multicellular organismal process and response to stress (Table S3). However, *NILR1* (*NEMATODE‐INDUCED LRR‐RLK 1*), the only reported receptor for ascr#18, showed no significant fold change compared to mock treatment (Table S3). Out of the 49 downregulated genes, four genes associated with auxin response and transport were found (Figure 4B,C). It included two genes, *IAA27* (*INDOLE‐3‐ACETIC ACID 27*) and *SAUR69* (*SMALL AUXIN UPREGULATED RNA 69*), families of which have been shown to play a pivotal role during early auxin responses (Luo et al. 2018; Stortenbeker and Bemer 2019), the *GH3.6* (*GRETSCHEN HAGEN 3.6*.) amido synthetase gene functioning in the production of auxin conjugates (Guo et al. 2022) and lastly, the auxin transporter *AUX1* (*AUXIN RESISTANT TRANSPORTER 1*), which has been suggested to play roles in root development and gravitropism (Marchant et al. 1999). Furthermore, RT‐qPCR analysis confirmed the downregulation of auxin signalling and transport genes following ascr#18 treatment (Figure 4D). Overall, RNA‐Seq analysis of ascr#18‐treated Arabidopsis plants revealed a modest transcriptional response, with notable downregulation of auxin transport and signalling genes, suggesting a specific modulation of auxin‐related processes rather than a broad immune response.

**FIGURE 5:**
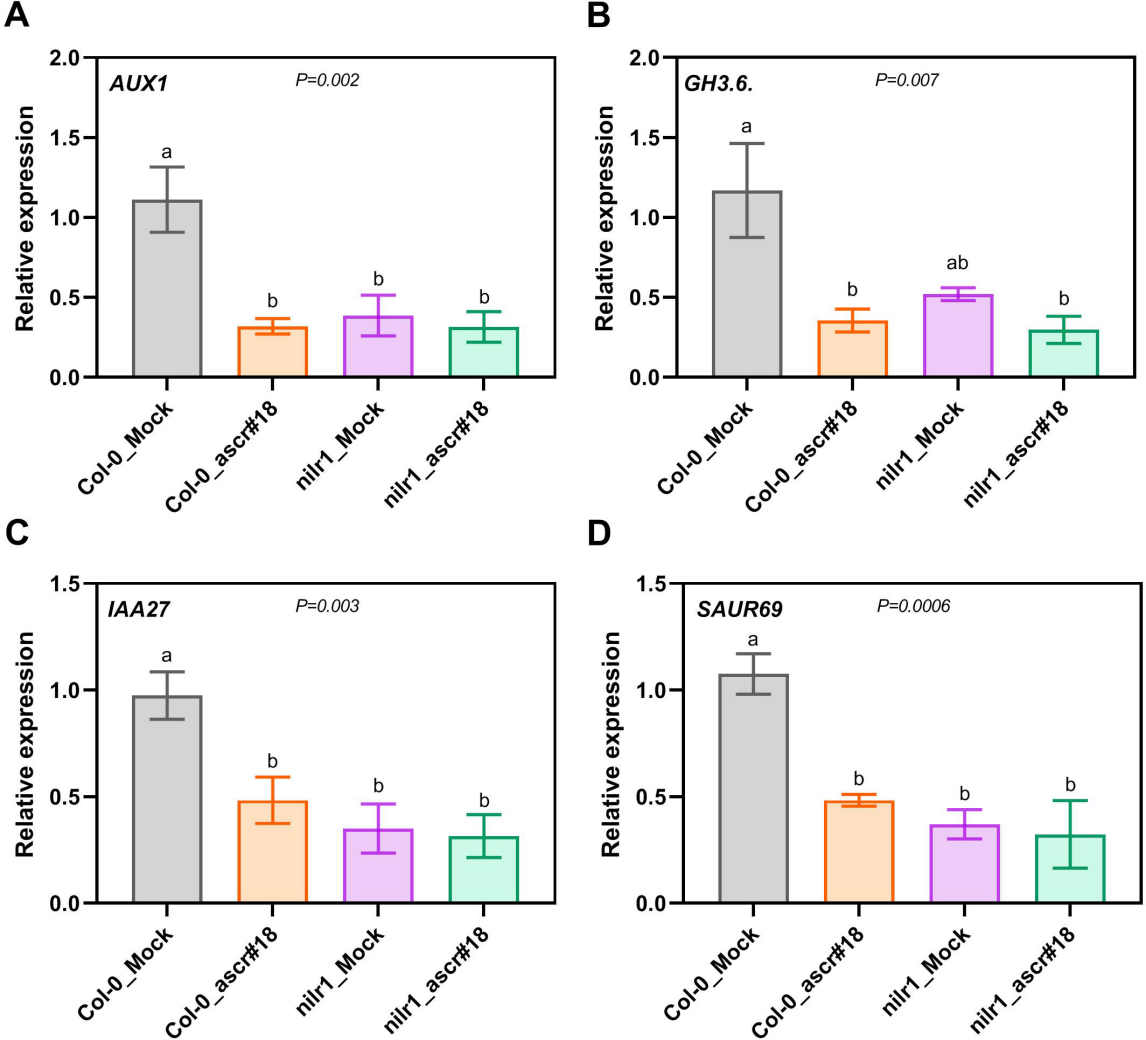
ascr#18‐induced auxin signalling suppression in Arabidopsis roots is independent of NILR1. Col‐0 and *nilr1* plants were treated with ascr#18 and sampled following the same protocol used for RNA‐Seq analysis. qPCR results show a downregulation of all four auxin‐related genes (AUX1, GH3.6, IAA27 and SAUR69) in both genotypes upon ascr#18 treatment. UBQ10 was used as an endogenous control. Each biological replicate represents the mean of three technical replicates, and final data represent the average of three biological replicates. Differences between means were analysed using one‐way ANOVA followed by Tukey's post hoc test. Statistically significant differences (*p* < 0.05) are indicated by different letters above the bars.

Next, to determine whether ascr#18‐induced auxin suppression in Arabidopsis roots depends on NILR1, the proposed receptor for ascr#18, we performed gene expression analysis using *nilr1* mutant plants. Our analysis revealed that all four auxin‐related genes (*AUX1*, *GH3.6*, *IAA27* and *SAUR69*) were downregulated upon ascr#18 treatment in both Col‐0 and *nilr1* plants (Figure 5). However, three out of four genes showed significantly reduced expression in *nilr1* plants regardless of ascr#18 treatment (Figure 5). These findings suggest that the ascr#18‐induced downregulation of auxin signalling genes is likely independent of *NILR1*.

### 3.5 Ascr#18‐Mediated Suppression of Auxin Transport and Signalling Persists During Nematode Infection

**FIGURE 6:**
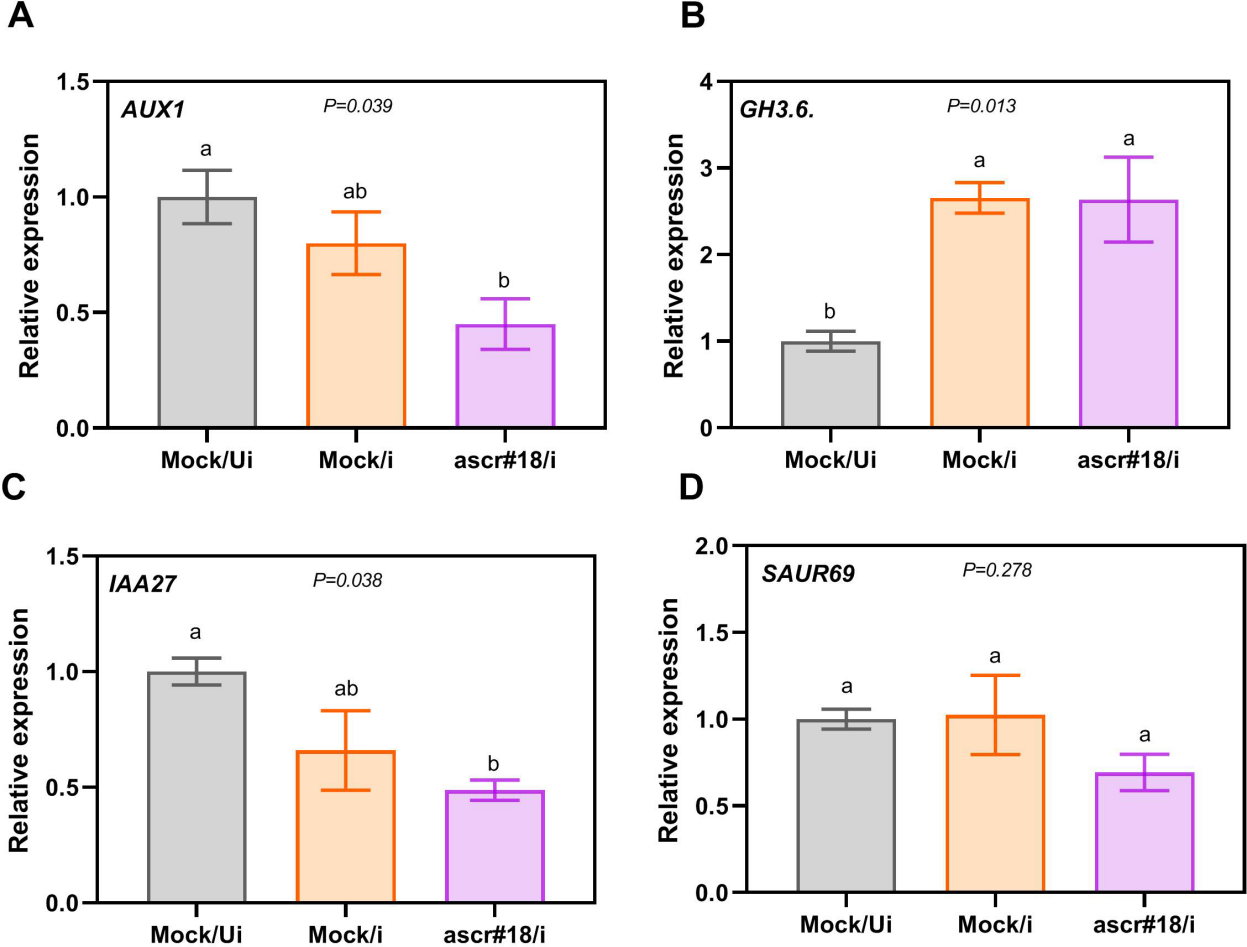
ascr#18 induces local auxin signalling suppression in infection sites. Expression analysis of auxin signalling genes showed downregulation of auxin transport and response genes in ascr#18 pretreated infection sites. Besides GH3.6, AUX1, 1AA27 and SAUR69 were downregulated in ascr#18‐pretreated*H. schachtii* infected sites in comparison to their water‐treated *H. schachtii*‐infected and water‐treated uninfected treatment groups. 12‐day‐old Col‐0 seedlings were treated with either water or ascr#18. At 6 h post treatment (hpt), the ‘infected’ category was inoculated with *H. schachtii* J2s. For each biological replicate, hundreds of tiny infected root sites (c.0.2 cm) were collected at three dpi and used for RNA extraction. Water‐treated uninfected root segments were used as a negative control. Bars represent mean ± SE for three independent repeats. Gene expression fold changes were calculated relative to the endogenous control of UBQ10, and the fold change for NC was set to 1. Letters above the bars indicate significant differences across treatments (*p* < 0.05; ANOVA). Mock/Ui, water‐treated uninfected roots; Mock/i, water‐treated infected sites; and ascr#18/i, ascr#18‐treated infected site.

RT‐qPCR validation confirmed the downregulation of auxin signalling genes observed in the RNA‐Seq data, and this downregulation is not linked to *NILR1*‐mediated downstream signalling. Notably, all analyses were conducted using samples independent of nematode infection. This raised a crucial question: does the ascr#18‐mediated suppression of auxin signalling persist during nematode infection? To address this, Col‐0 plants were pretreated with 1 mL/plant of either 1 μM ascr#18 or sterile water for 6 h before inoculation with *H. schachtii* J2s. At 3 dpi, nematode infection sites were collected, with uninfected water‐treated Col‐0 plants serving as mock controls. RT‐qPCR analysis revealed that three of the four target genes were downregulated in ascr#18‐treated infected samples, with *AUX1* and *IAA27* showing significant reduction (Figure 6A,C,D). By contrast, *GH3.6* exhibited significant upregulation in infected samples regardless of pretreatment (Figure 6B). These results demonstrate that ascr#18 pretreatment maintains its suppressive effect on auxin transport and signalling genes even during nematode infection.

### 3.6 Ascr#18 Suppresses AUX1‐Mediated Auxin Transport During the Early Stage of Syncytium Development by *H. schachtii*

**FIGURE 7:**
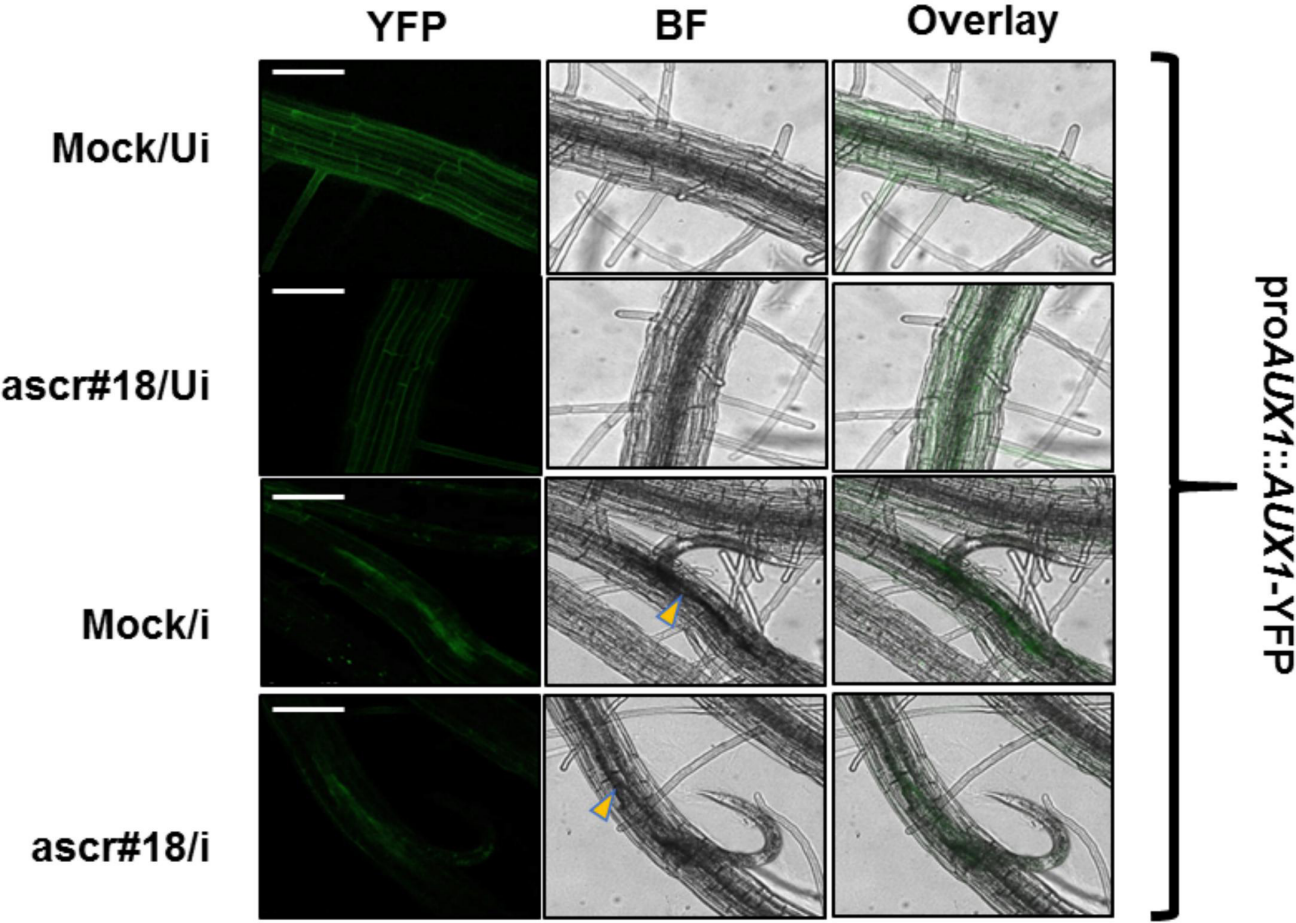
ascr#18 triggers a reduction in *AUX1* gene expression in plant roots, particularly at nematode feeding sites. (a) Representative confocal images of pro*AUX1*::*AUX1*‐YFP expression in *A. thaliana* roots upon nematode infections (3 dpi) with or without ascr#18 treatment. Approximately five infected plants per treatment per biological replicate were imaged, and the experiment was repeated three times. Scale bars = 100 μm. ‘YFP’—Yellow fluorescent protein, ‘BF’—Bright field, ‘Ui’—Uninfected, ‘i’—Infected. Arrowheads indicate infection point.

The auxin transport system, particularly the *AUX1* influx carriers, is essential for gall formation during early root‐knot nematode (RKN) infection in *Arabidopsis* (Kyndt et al. 2016). Our gene expression analysis revealed that ascr#18 disrupts auxin transport during early syncytium development by suppressing *AUX1* expression. To visualise AUX1's spatial distribution in *H. schachtii* ‐induced syncytia following ascr#18 treatment, we employed an Arabidopsis line expressing an AUX1‐YFP fusion protein under its native promoter (Swarup et al. 2004). We examined four experimental conditions: (1) untreated, uninfected roots (mock/Ui), (2) ascr#18‐treated, uninfected roots (ascr#18/Ui), (3) untreated, infected roots at 3 dpi (mock/i) and (4) ascr#18‐treated, infected roots (ascr#18/i). As shown in representative pictures in Figure 7, ascr#18 treatment reduced constitutive AUX1‐YFP fluorescence in uninfected roots compared to mock controls, demonstrating that ascr#18 alone suppresses *AUX1* expression. While *AUX1* expression was locally induced in young syncytia at 3 dpi, indicating active auxin influx during early syncytium development, this expression was significantly reduced in ascr#18‐treated syncytia. Intriguingly, it could also be observed that under uninfected conditions, with and without ascr#18 treatment, the YFP signal was distinctively distributed over the root vasculature, particularly within the epidermal cells, which diffused gradually as the infection started. This result demonstrates that ascr#18 interferes with auxin transport in uninfected roots and nematode feeding sites during cyst nematode infection by downregulating *AUX1* expression.

Next, to investigate whether auxin availability to the developing syncytium is reduced upon ascr#18 treatment, we analysed DR5::GUS reporter lines with and without ascr#18 exposure. Our analysis revealed that GUS intensities were notably reduced at nematode infection sites at 3 dpi in ascr#18‐treated plants compared to mock‐treated controls (Figure S6). A similar reduction was also observed in root tip staining (Figure S6). These results provide direct evidence of reduced auxin availability in the developing syncytium, likely due to ascr#18‐induced suppression of auxin signalling genes.

## 4 Discussion

Nematode pheromone belonging to the class of ascaroside glycolipids was identified as the first NAMP present across diverse PPN species, including sedentary genera (*Meloidogyne* and *Heterodera*) and migratory ones (*Pratylenchus*). Among the identified ascarosides, ascr#18 was most abundant and demonstrated the capacity to induce immune responses, including upregulation of defence marker genes FRK1, AOS and PR4 (Manosalva et al. 2015). The cognate PRR for ascr#18 remained unknown until 2023, when Huang et al. (2023) reported that ascr#18 binds to the ligand‐binding domain of *NILR1*, a leucine‐rich repeat nematode‐associated PRR. This finding contradicted our earlier work showing *NILR1* likely recognises a proteinaceous ligand (Mendy et al. 2017). In addition, insights from the resolved crystal structure of the *NILR1* extracellular domain support an alternative model, in which *NILR1* is more suited to perceive a small‐molecule ligand rather than larger macromolecules such as peptides, nucleic acids, or lipids (Wu et al. 2017). The ascr#18‐*NILR1* interaction raises questions due to discrepancies between previously reported *nilr1* responses and our current findings with ascr#18. While Mendy et al. (2017) demonstrated that *NILR1* loss‐of‐function compromised typical PTI responses, including ROS burst and growth inhibition upon NemaWater treatment, our results show that ascr#18 fails to induce a typical defence‐related ROS burst compared to the bacterial PAMP flg22. The ROS levels in ascr#18‐treated Col‐0 plants at different ascaroside concentrations remained comparable to the water control, even after medium (3 h) and long‐term (6 h) exposure. Our results tally with previous work done by Ning et al. (2020), who reported that Arabidopsis leaves treated with ascr‐#1, #5, #10 and #18 did not induce ROS burst. Jointly, these two findings confirm the absence of ROS bursts as an immune response upon the plant's exposure to ascarosides, irrespective of the ascaroside species, the ascr#18 concentrations used, and the plant's exposure time to the NAMP. Additionally, ascr#18 pretreatment of Col‐0 plants did not trigger canonical growth inhibition, typically considered a hallmark of PTI activation and viewed as an energy trade‐off for immune responses against non‐self‐threats (Karasov et al. 2017; Huang, Yuan, Ramirez, Zhao, et al. 2024). These atypical defence patterns induced by ascr#18, coupled with the contrasting PTI responses previously reported in *nilr1* mutants, raise questions about the ascr#18‐*NILR1* dynamic and suggest the existence of non‐canonical ascr#18‐mediated defence mechanisms.

In lieu of alternative mechanisms, Manohar et al. (2020) demonstrated that Arabidopsis metabolically converts ascr#18 to a shorter‐chain variant, ascr#9, via the peroxisomal β‐oxidation pathway, which acts as a nematode repellent against *Meloidogyne incognita* and constitutes an independent defence response. However, our analysis indicates that this metabolic pathway may not be the sole contributor to ascr#18‐related defence. The *acx1acx5* double mutant, which lacks this β‐oxidation pathway, does not exhibit compromised resistance to *H. schachtii* or altered ROS responses, despite elevated internal levels of ascr#18. Notably, ascr#18 still induced resistance in *acx1acx5* plants, as shown by reduced infection rates as well as smaller female nematodes and syncytia in ascr#18‐pretreated plants compared to mock controls. These findings suggest the presence of an additional, previously unrecognised ascr#18‐mediated defence mechanism in *A. thaliana* that functions independently of metabolic editing and is distinct from classical PTI responses. Supporting this notion, ascr#18 also elicits immune responses against a wide range of pathogens, including *Pseudomonas syringae* DC3000 (Klessig et al. 2019; Huang et al. 2023); however, its metabolic conversion does not appear to contribute to resistance against this bacterial pathogen (Manohar et al. 2020), indicating that the metabolic editing mechanism may be specific to the Arabidopsis– *M. incognita* pathosystem. This prompted us to pursue RNA‐Seq analysis to uncover the broader landscape of ascr#18‐associated defence responses. Interestingly, our transcriptome profiling, conducted using the commonly applied micromolar concentration (1 μM) of ascr#18 with water as the solvent and a 6‐h treatment duration, revealed no induction of typical defence‐related genes, nor was the expression of *NILR1* altered. In contrast, Huang et al. (2023) initially dissolved ascr#18 in DMSO and observed upregulation of several defence‐related genes 48 h after treatment with 10 nM ascr#18. Similarly, Manohar et al. (2020) reported the induction of defence‐response genes in Arabidopsis leaves following root treatment with 1 μM ascr#18, which was initially dissolved in ethanol. However, transcriptome profiling at nanomolar concentrations in the same study did not reveal activation of defence‐related genes, presumably including *NILR1*. These contrasting findings emphasise the need for a standardised approach in ascr#18 research. Although nanomolar concentrations of ascr#18 do not induce defence genes in transcriptomic analyses, as reported by Manohar et al. (2020), these same concentrations, particularly 10 nM, have been shown to be effective against *Phytophthora infestans* and *Pseudomonas syringae* in tomato, as well as *Blumeria graminis* in barley (Manosalva et al. 2015), suggesting the involvement of an alternative defence mechanism.

Surprisingly, transcriptomic analysis of ascr#18‐treated plant roots revealed downregulation of several auxin transport and signalling genes (*IAA27*, *SAUR69*, *GH3.6* and *AUX1*). This reduced expression could explain the decreased size of *H. schachtii* ‐induced syncytia and smaller female nematodes in ascr#18‐treated plants, considering auxin's essential role in nematode feeding site formation (Goverse et al. 2000; Karczmarek et al. 2004; Kyndt et al. 2016; Siddique et al. 2022). Gene expression analysis of young syncytia (3 dpi) from plants pretreated with ascr#18 before *H. schachtii* inoculation demonstrated significant downregulation of *AUX1* and *IAA27*. *SAUR69* showed consistently lower expression in ascr#18‐pretreated‐ *H. schachtii* ‐infected plants compared to water‐ *H. schachtii* ‐treated controls, though this difference did not reach statistical significance across biological replicates. This pattern may be explained by the endogenous presence of ascr#18 in the *Heterodera* genus, albeit at lower concentrations (Manosalva et al. 2015), suggesting a concentration‐dependent effect where exogenous ascr#18 application enhances the suppression of auxin signalling. These findings confirm the downregulation of auxin signalling genes in the nematode feeding sites in response to ascr#18 exposure. Interestingly, *GH3.6*, encoding an auxin‐conjugating enzyme, is downregulated upon ascr#18 treatment, likely to suppress auxin signalling, similar to *AUX1*, *IAA27* and *SAUR69*. However, unlike these genes, *GH3.6* is upregulated in nematode‐induced syncytia, irrespective of ascr#18 treatment. This upregulation is likely a host response to buffer excess auxin and maintain hormonal balance in response to the high metabolic activity and cellular reprogramming at the feeding site. Notably, *GH3* genes are generally induced during pathogen infection, supporting their role in stress‐related auxin regulation (Hui et al. 2019). The downregulation of auxin signalling genes in *nilr1* plants, regardless of ascr#18 treatment, suggests that ascr#18‐induced downregulation of these genes is likely independent of *NILR1*. However, as a receptor, *NILR1* may still be involved in broader regulatory networks related to plant hormone signalling, including auxin, particularly given its previously demonstrated role in brassinosteroid signalling (Zheng et al. 2022). A comprehensive understanding of the signalling pathways downstream of *NILR1* is beyond the scope of this study, but this area is highlighted as an important avenue for future research.

In our promoter‐reporter analysis using the *AUX1‐YFP* transgenic Arabidopsis line, we observed that *AUX1* expression was locally induced in young syncytia at 3 dpi, suggesting active auxin influx during early syncytium development. However, this expression was significantly reduced in ascr#18‐treated syncytia, indicating a potential disruption of auxin dynamics. The role of auxin as a virulence factor has been well‐documented in both hemibiotrophic and biotrophic pathogens. For example, Kyndt et al. (2016) demonstrated that sedentary nematodes manipulate auxin transport to facilitate feeding site formation. They showed that a delicate balance between auxin influx (via *AUX1*) and efflux (via *PIN1*) is crucial for normal giant cell and gall development. In *A. thaliana* , the *aux1* mutant, which lacks proper auxin influx, produced smaller galls compared to the Col‐0 and exhibited transgenerational developmental defects, including the absence of mature female nematodes with eggs. Similarly, Goverse et al. (2000) found that *A. thaliana* auxin‐insensitive mutants (*axr* and *aux1*) displayed increased resistance to the beet cyst nematode, *H. schachtii* , with smaller syncytia observed in the mutants compared to the Col‐0. The role of auxin transport in nematode parasitism was further confirmed in tomato plants pretreated with N‐(1‐naphthyl)phthalamic acid (NPA), a chemical that disrupts auxin homeostasis. NPA‐treated tomato plants showed delayed nematode development and a 60% reduction in nematode infection compared to untreated plants, reinforcing the critical role of auxin in nematode parasitism (Goverse et al. 2000). These findings support the hypothesis that ascr#18 may interfere with auxin transport, potentially influencing nematode feeding site formation in *A. thaliana* .

The role of auxin in facilitating successful biotrophic fungal parasitism has been extensively studied. Several species of smut fungi, for instance, have been shown to produce their own auxin. *Ustilago maydis* , for example, produces auxin through an NAD‐dependent pathway, while the head smut fungus *Sporisorium scitamineum* carries a gene responsible for coding tryptophan aminotransferase, which catalyses the first step in tryptophan‐dependent auxin production (Nagarajan et al. 2023). Interestingly, Reineke et al. (2008) demonstrated that mutations in the auxin biosynthesis pathway of *U. maydis* reduced auxin levels in the host, but gall formation remained comparable to that in wild‐type‐infected plants. This suggests the involvement of additional mechanisms, such as host auxin transcriptional modification by the biotrophic fungi. Supporting this hypothesis, Doehlemann et al. (2008) reported the upregulation of 3 auxin biosynthesis genes and 19 auxin response genes in maize inoculated with *U. maydis* as early as 4 dpi, further indicating a complex interaction between the host and the pathogen that modulates auxin‐related pathways.

In hemibiotrophic pathogens like *Pseudomonas syringae* strain DC3000, auxin production has been shown to facilitate parasitism. The bacterium produces its own auxin via indole‐3‐acetaldehyde dehydrogenase (AldA), and the loss of function of *AldA* impairs its ability to produce auxin in the host, resulting in reduced virulence compared to the wild‐type strain (McClerklin et al. 2018). Additionally, exogenous auxin application has been shown to enhance disease proliferation, which is partly due to crosstalk between auxin and salicylic acid (SA). The relationship between these two hormones is inversely proportional, with auxin repressing SA‐mediated defences and vice versa. For instance, plants inoculated with *P. syringae* lacking the *AldA* gene showed higher expression of *PR1*, a SA‐related defence gene, indicating that auxin can suppress SA‐dependent defence responses. Furthermore, silencing auxin signalling through inhibition of the auxin receptor, *AXR2‐1*, resulted in increased susceptibility to *P. syringae* , and this susceptibility was reversed by the introduction of the SA mutant *sid2‐2* in the *axr2‐1* background, confirming the antagonistic interaction between auxin and SA (Djami‐Tchatchou et al. 2020; McClerklin et al. 2018; Wang et al. 2007). Auxin also directly influences bacterial virulence by inducing the expression of other virulence factors in both culture and in planta (Djami‐Tchatchou et al. 2020). These findings collectively highlight the role of auxin as a susceptibility and virulence factor that promotes parasitism in a wide range of pathogens. It is, therefore, not surprising that plants have evolved defence mechanisms aimed at suppressing auxin signalling. One such mechanism involves the post‐transcriptional degradation of auxin signalling mRNAs via the silencing machinery. For example, *Arabidopsis thaliana* recruits microRNA miR393a to degrade the mRNA of auxin signalling genes, such as *GH3‐like*, *IAA12* and *IAA17*, as early as 1.5 h after perception of the PAMP flg22 (Navarro et al. 2006). Overexpression of miR393 in the wild‐type background led to constitutive suppression of auxin signalling and resulted in five times higher bacterial growth compared to wild‐type plants. Interestingly, these same auxin signalling genes were downregulated in both RNA‐Seq and RT‐qPCR analyses upon treatment with *ascr#18* and nematode infection in *A. thaliana* (Navarro et al. 2006). A recent study also showed that potato employs RNA metabolism to post‐transcriptionally downregulate a cyclin gene critical for syncytium development, thereby enhancing basal resistance to the potato cyst nematode, *Globodera pallida* (Huang, Yuan, Ramirez, Xia, et al. 2024). This raises the question: Could the si‐miRNA machinery be responsible for the observed downregulation of auxin signalling genes in response to *ascr#18* treatment?

**FIGURE 8:**
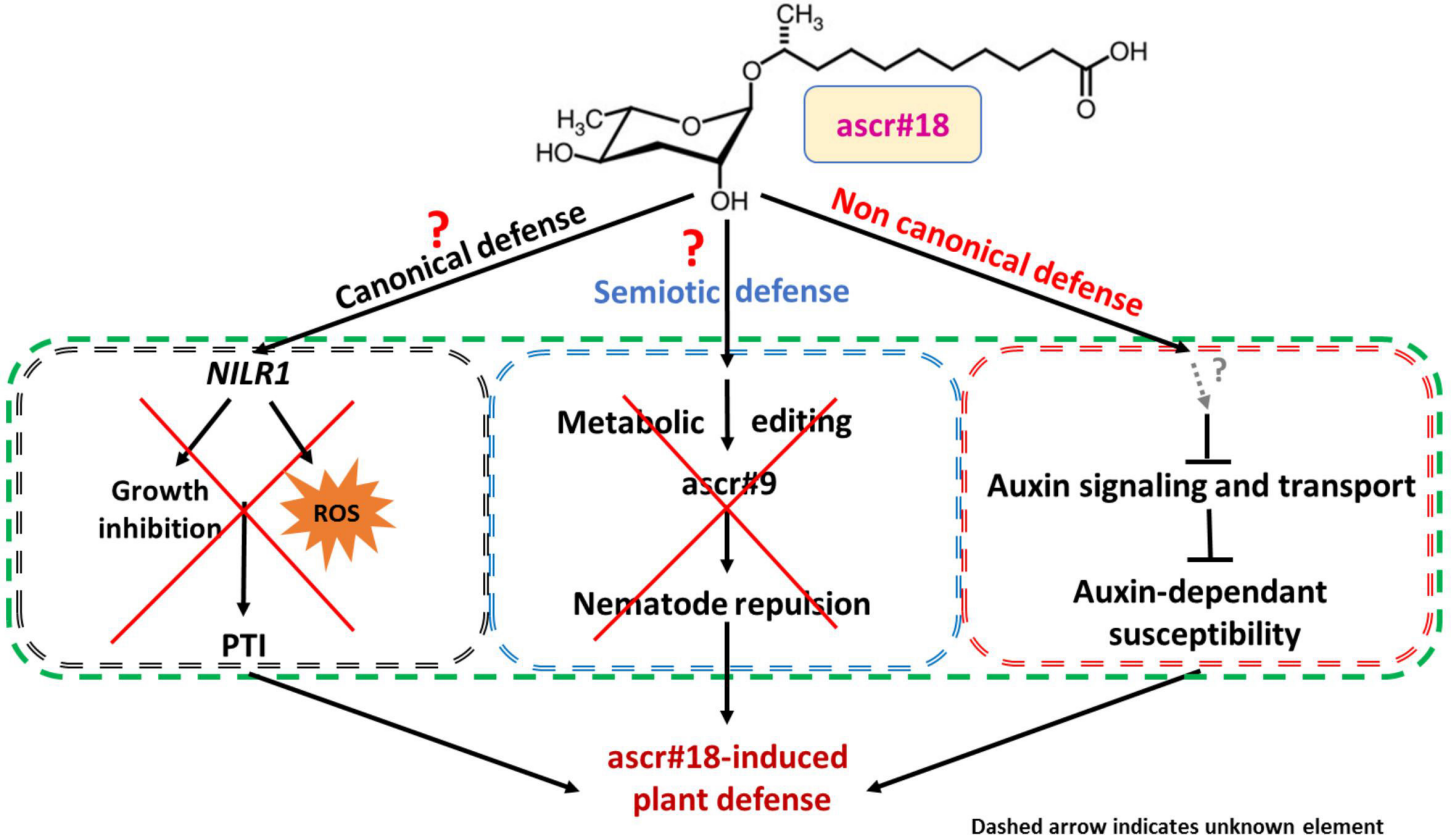
ascr#18‐dependent non‐canonical plant resistance to cyst nematodes. Our study reveals that ascr#18 mediates a non‐canonical form of resistance in *Arabidopsis* roots against nematodes. Notably, ascr#18 does not induce canonical pattern‐triggered immunity (PTI) responses such as oxidative ROS bursts, defence‐related seedling growth inhibition, defence gene expression, or transcription of *NILR1* **—**the only reported NAMP receptor gene. This indicates a unique molecular interaction that bypasses typical plant defence pathways, contrasting with findings reported by Manosalva et al. (2015) and Huang et al. (2023). Also our data do not support the non‐cellular semiotic defence dependent on metabolic editing, as described by Manohar et al. (2020). Instead, we propose that ascr#18 triggers the downregulation of auxin transport and signalling in *Arabidopsis* roots through an as‐yet‐undefined mechanism. This suppression of auxin signalling may play a critical role in limiting nematode infection and impairing the development of cyst nematodes and their feeding sites.

While we do not yet have experimental evidence to support this hypothesis, several studies have explored the potential of *ascr#18* as a broad‐spectrum biocontrol agent, demonstrating its ability to confer defence against various viral, fungal and bacterial pathogens (Akshita et al. 2024; Klessig et al. 2019; Manosalva et al. 2015). Of particular interest are studies that have shown that pretreating plants with *ascr#18* can specifically confer resistance to *Pseudomonas syringae* (Huang et al. 2023; Manosalva et al. 2015). Given our findings (Figure 8), it is plausible that *P. syringae* struggles to parasitise plants pretreated with ascr#18 due to the suppression of auxin signalling, which we report here. The downregulated genes in our analysis belong to the same gene family as those identified by Navarro et al. (2006), leading us to hypothesise that *ascr#18* exposure triggers a series of events that lead to the downregulation of auxin signalling in *Arabidopsis*, potentially through an as‐yet‐undefined mechanism. This suppression of auxin signalling may contribute to the broad‐spectrum effectiveness of *ascr#18* as a biocontrol agent against a variety of pathogens. To the best of our knowledge, this is the first evidence suggesting that suppression of auxin signalling constitutes an independent defence mechanism associated with *ascr#18* in plants.

## Author Contributions

M.S.H., M.I. and F.M.W.G. conceptualised the study. S.L., S.B., B.M., S.H.v.R. and M.S.H. performed experiments. S.L., S.B., U.C.V. and M.S.H. analysed the data. S.L., S.B. and M.S.H. curated the data and performed the statistics. S.L. and M.S.H. wrote the original draft. All authors contributed to the editing of the manuscript.

## Conflicts of Interest

The authors declare no conflicts of interest.

## Supporting information


**Data S1.** Supplementary Information.

**Table S2.** Total readcounts and mapping statistics of the samples used for RNA‐Seq used in this study.

**Table S3.** All the DEGs obtained with ascr#18 vs. Mock, at FDR < 0.05.

## Data Availability

All data are available in the main text or the Supporting Information. Raw RNA‐Seq data used in this study has been made publicly available on the Sequence Read Archive (SRA) database from NCBI, with the following ID: PRJNA1191218.
