# Supplementary material for: Ascaroside#18 Promotes Plant Defence by Repressing Auxin Signalling
Source: Physiol Plant. 2025 Jul 11;177(4):e70386. doi: 10.1111/ppl.70386 (PMC12247022; doi:10.1111/ppl.70386)
Supplement: Supplementary file 1 — Data S1. Supplementary Information. [file PPL-177-e70386-s001.docx]

**Supplementary materials:**

| **A.** | **B.** |
| --- | --- |
| **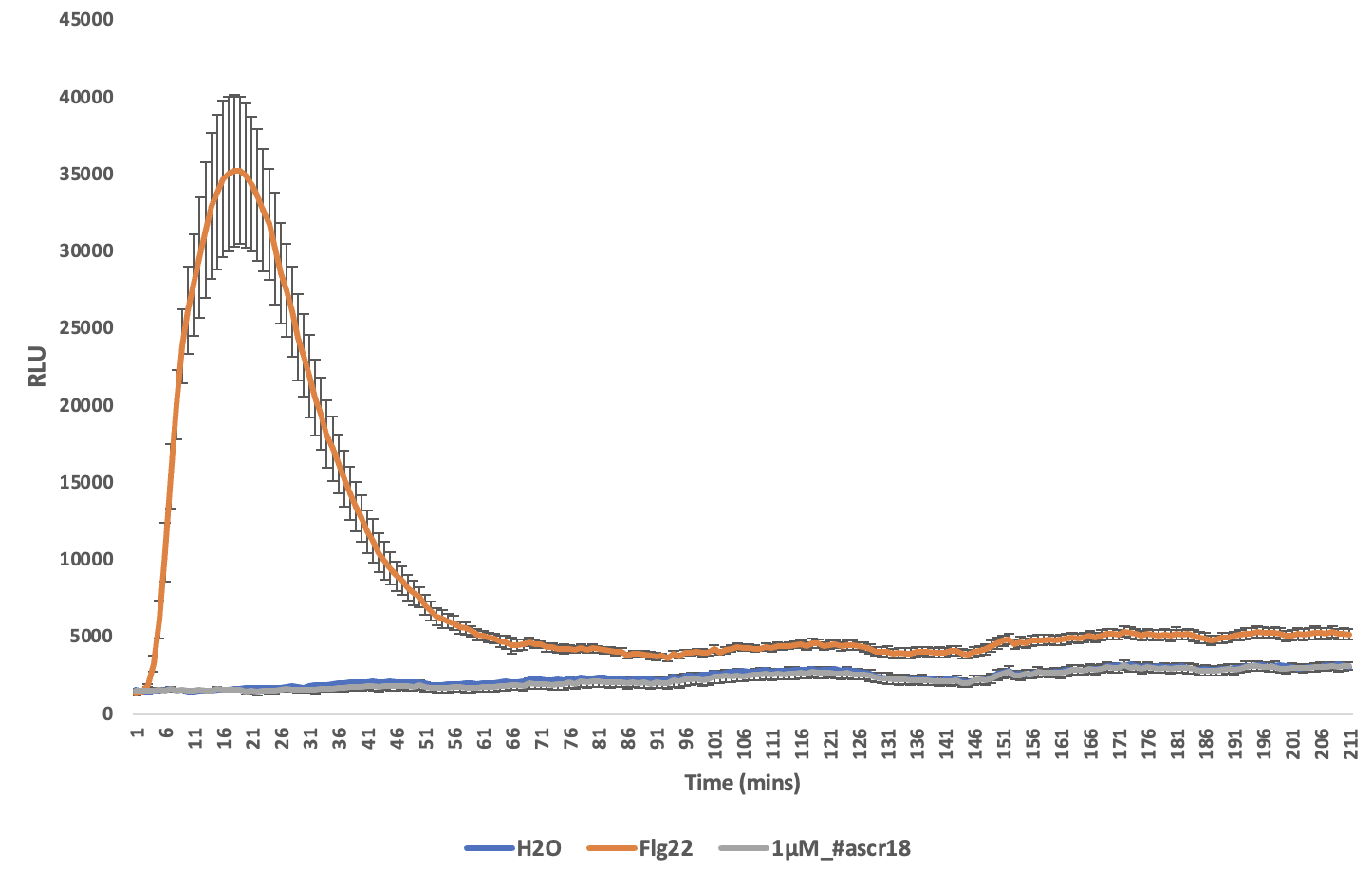** | **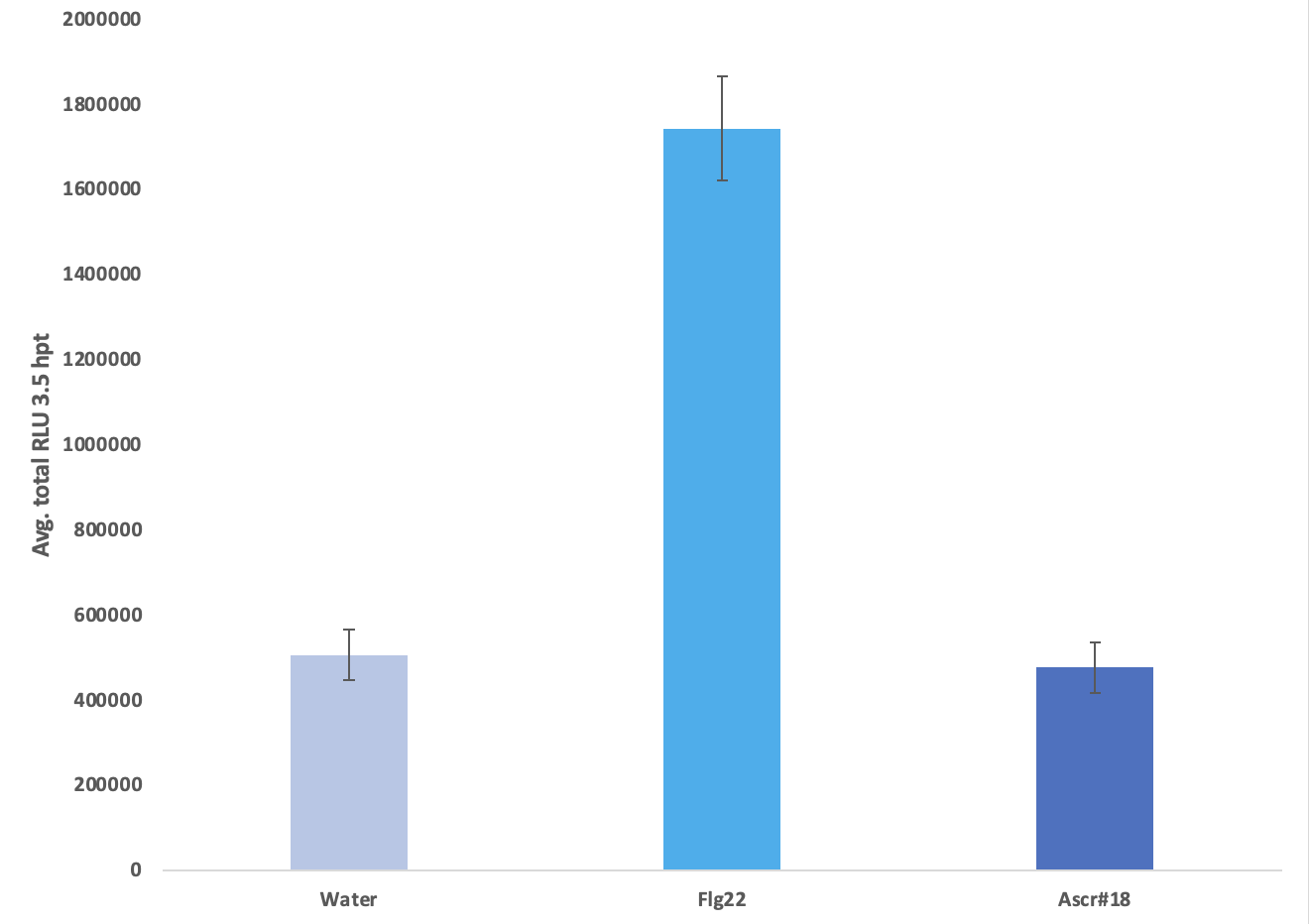** |
| **C.** | **D.** |
| **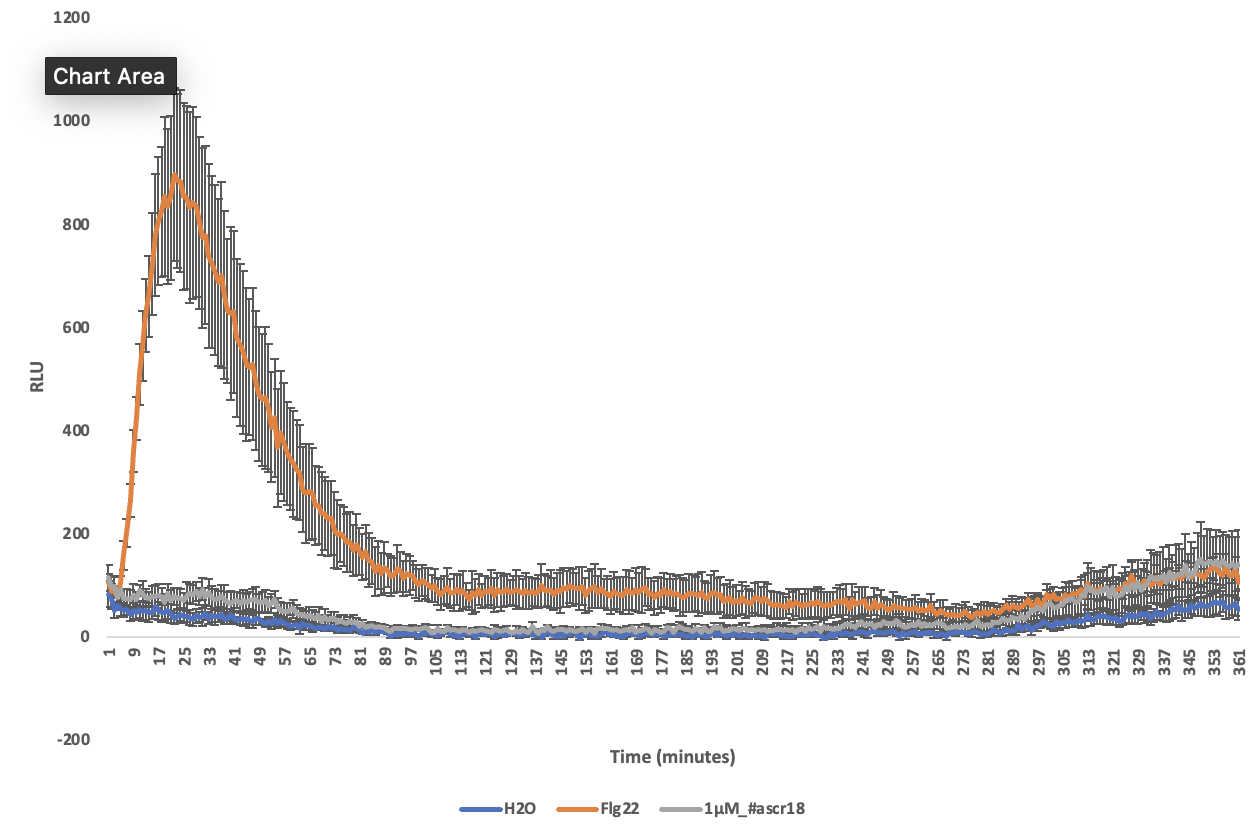** | **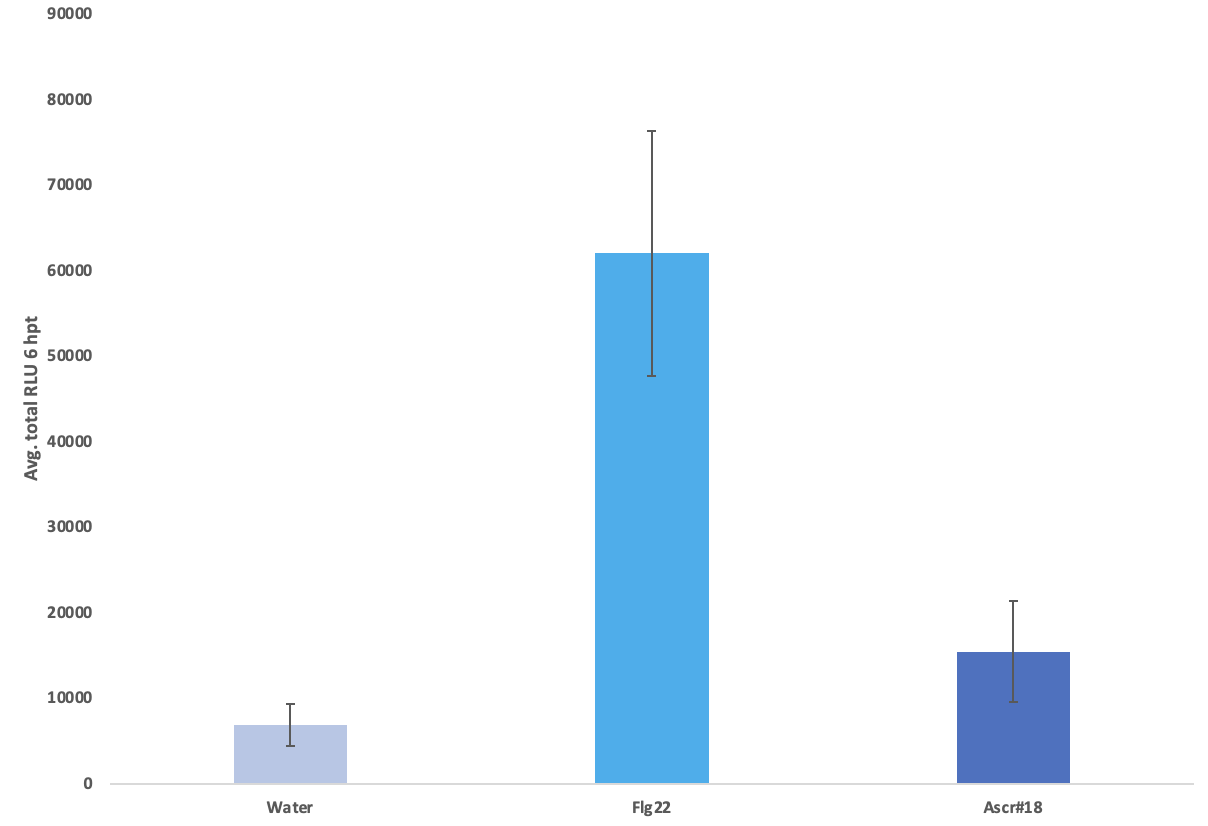** |
| **Supplementary Figure S1: Ascr#18 does not induce significant ROS bursts even after long periods of treatment.** Col-0 leave discs were cut and treated with water, ascr#18 and bacterial peptide Flg22 was used as positive control. ROS bursts in the form of relative light units (RLU) was measured using the luminol-based assay. A-B: ROS production measured over 3.5 hours post treatment (3.5 hpt) and total ROS produced in the same time respectively. C-D: ROS production measured over 6 hours post treatment (6hpt) and total ROS produced in the same time respectively. For both exposure times, Flg22 triggered typical defense-related ROS bursts but ascr#18 had no burst. Total ROS produced by ascr#18 over the course of the experiment was comparable to that produced by water. The mean ±SE of 3 technical replicates was plotted per minute. Experiment was repeated 3 times independently yielding the same results. Letters above bars represent statistical differences between treatments (P<0.05, ANOVA).    **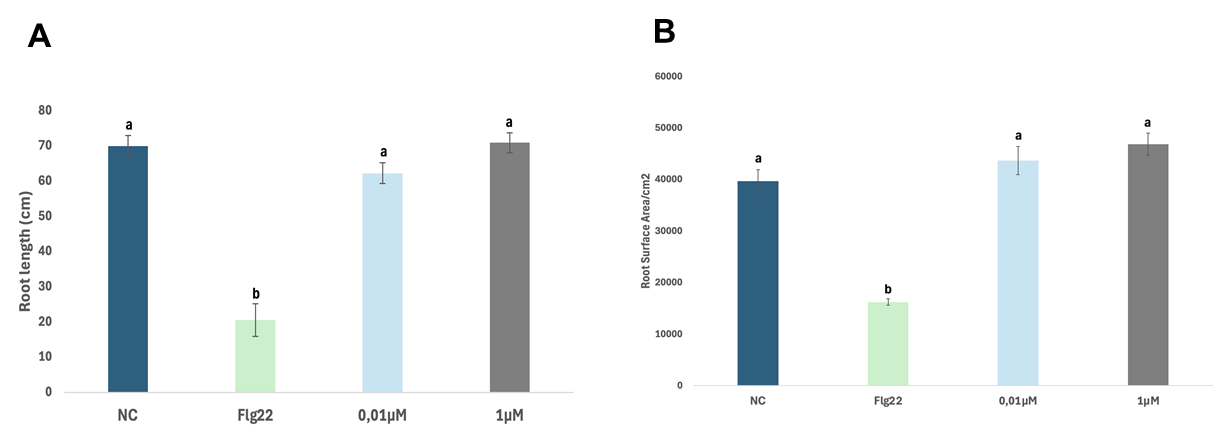**  **Supplementary Figure S2:** **Ascr#18 does not induce typical growth inhibition upon its perception.** Five-day old Col-0 seedlings were transplanted into 6-well plates containing water, ascr#18 and Flg22, root length and root surface area was measured at 12 dps; 7 days post transplanting. Root length and root surface area in Flg22-treated plants was significantly lower than that of the ascr#18-treated and water-treated plants; seedlings treated with ascr#18 did not exhibit any growth inhibition. A, average seedling root length measured at 7 days post treatment (7 dpt). B, average seedling root surface area at 7 dpt. Root images for phenotyping root length and root surface area were obtained using the Epson root scanners and measurements were done using the WinRhizo application. Each treatment had 12 plants per biological repetition. Experiment was repeated on 3 independent occasions. Bars represent means ±SE, n=12 and letters show significant differences (P<0.05, ANOVA).  **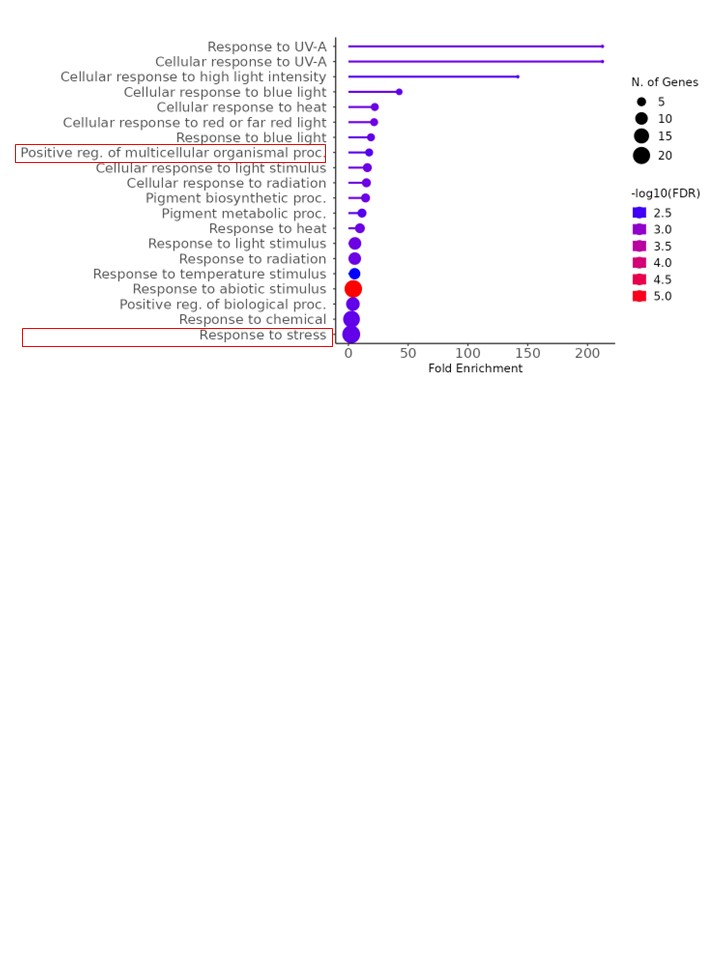**  **Supplementary Figure S3: Top 20 biological process related GO terms associated with the up-regulated genes obtained in this study.**  **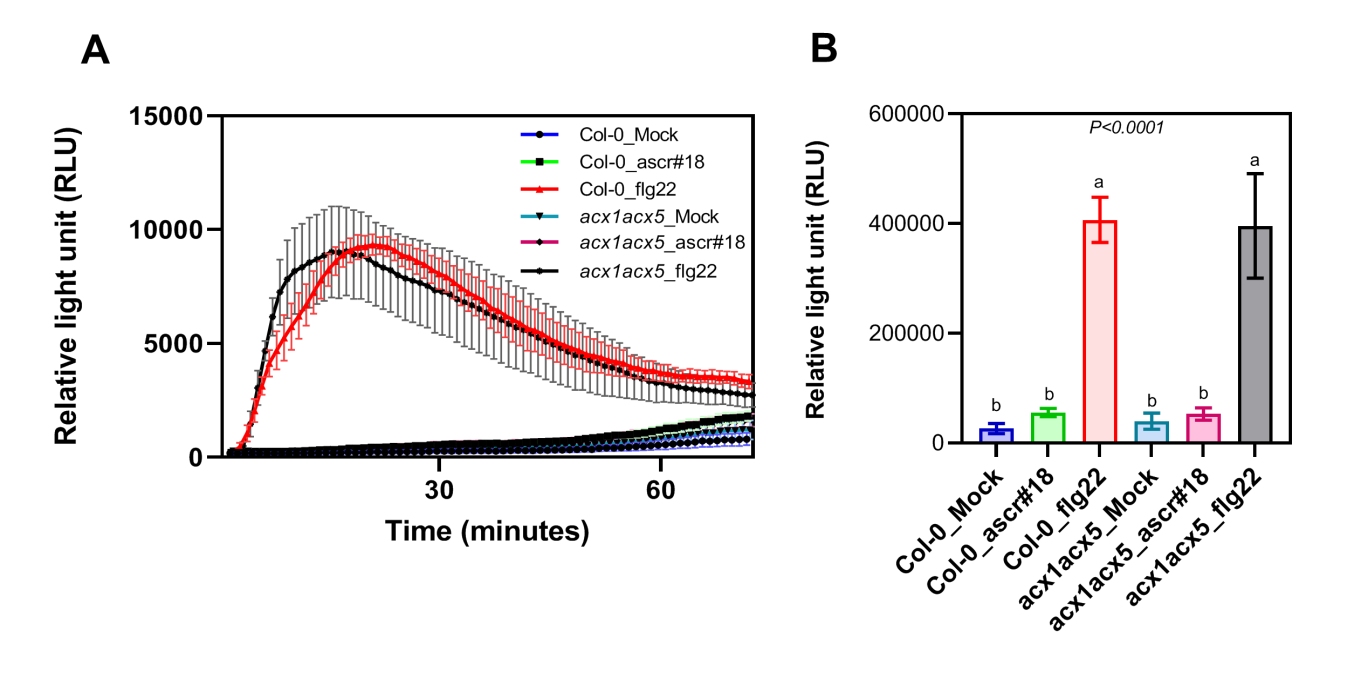**  **Supplementary Figure S4: Elicitor induced ROS bursts is unchanged in acx1acx5 plants.** A. Leaf discs were cut and treated with water, 1 μM ascr#18, and flg22 as a positive control. ROS bursts, measured as relative light units (RLU), were monitored using a luminol-based assay over 90 minutes. Both Col-0 and *acx1acx5* plants exhibited flg22-triggered ROS bursts, while no ascr#18-induced ROS burst was observed in either genotype. Data represent the mean ± SE of three biological replicates, recorded per minute. The experiment was independently repeated three times. B. Total ROS production over 90 minutes post-elicitor treatment. The total ROS produced in response to flg22 was statistically similar between *acx1acx5* and Col-0 plants. Likewise, the ascr#18-induced ROS levels in *acx1acx5* were comparable to those in Col-0. Ascr#18 treatment consistently showed no significant ROS induction compared to the mock control throughout the experiment. Letters above the bars indicate statistically significant differences between treatments (P < 0.05, ANOVA).  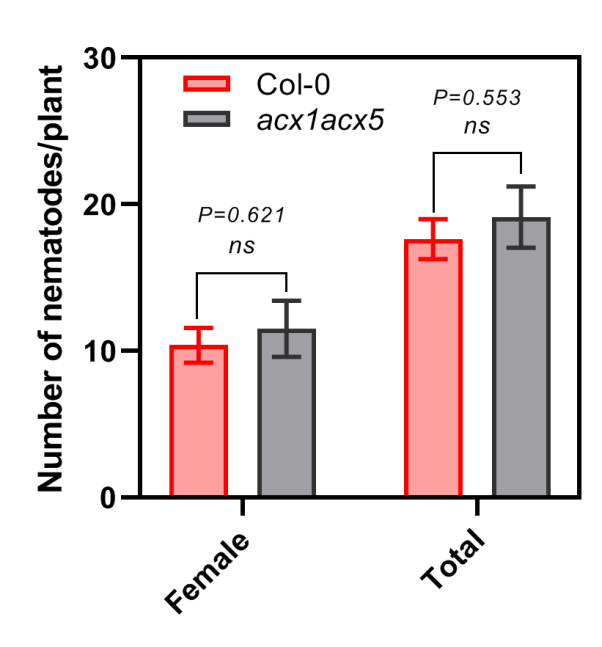  **Supplementary Figure S5: Infection assays of *acx1acx5* plants with *H. schachtii*.** The β-oxidation loss-of-function double mutant *acx1acx5* showed no infection phenotypes to *H. schachtii*. Results were consistent across at least three independent experiments, with one representative dataset presented. Bars indicate mean ± SE; statistical significance was assessed using a *t*-test.  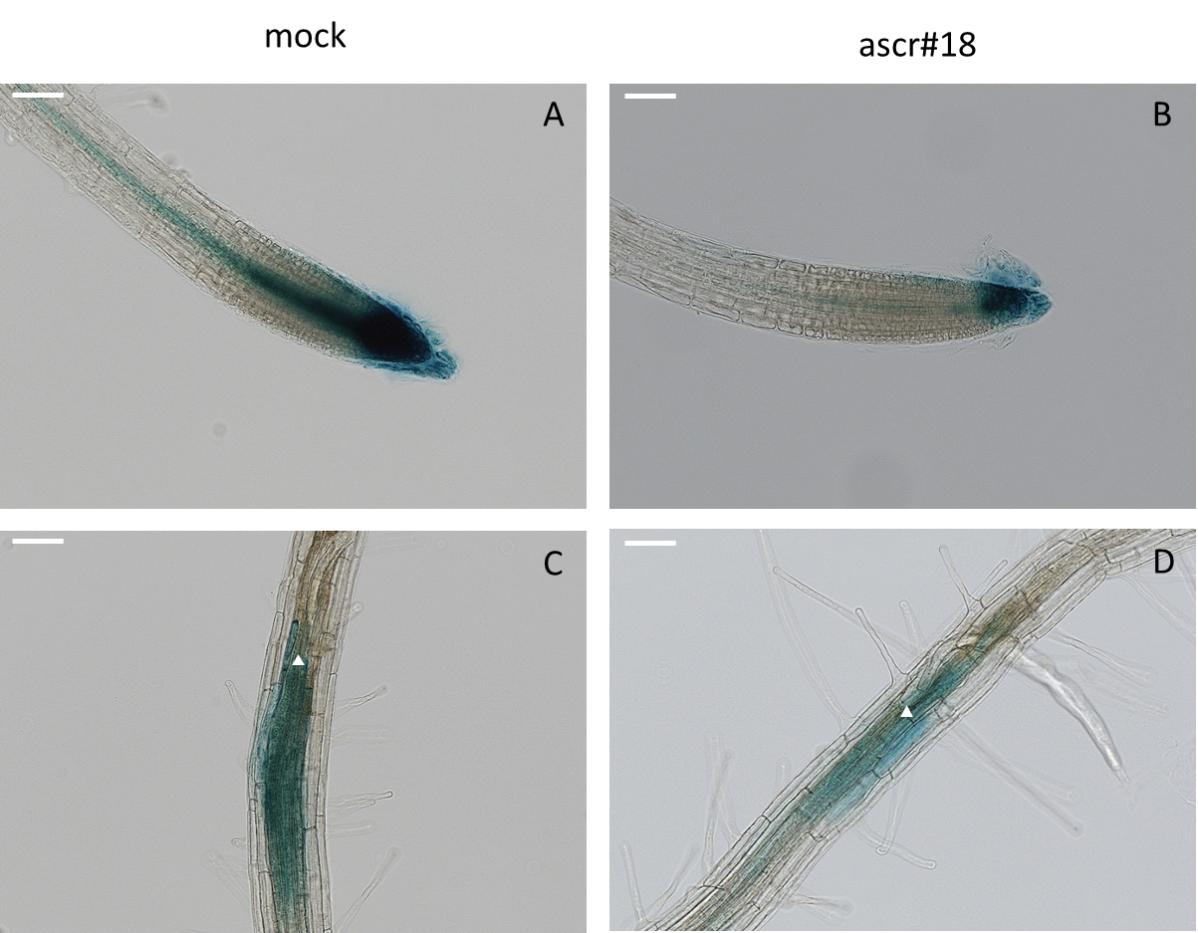  **Supplementary Figure S6:** **DR5:GUS expression at 3 days post inoculation (dpi) upon ascr#18 and Mock treatment**. A-B, DR5::GUS expression in the root tips of mock and ascr#18 treated plants. C-D, DR5::GUS expression at the nematode infection sites at 3 dpi of mock and ascr#18 treated plants. Arrowheads indicate nematodes. Bar, 50 µm. | |

**Supplementary Table S1: List of gene sequences used for RT-qPCR**

| Genes | Genes |
| --- | --- |
| F_qGH3.6 | CACCACCTATGCTGGGCTT |
| R_qGH3.6 | CAAGGTGTGTTACCGCGTTTT |
| F_qSAUR69 | GACCGTTGGTTATCCCGTGT |
| R_qSAUR69 | CCGGTCACCAGATGATAGCC |
| F_qAUX1 | GCCTCCGCTCGTCAGAAT |
| R_qAUX1 | ACGGTGGTGTAAAGCGGAGA |
| F_qIAA27 | AGCTCCAGCTTCAAAAGCAC |
| R_qIAA27 | GGTCCAGACTTAGCTTCTGCT |
| F_18 S rRNA | GGTGGTAACGGGTGACGGAGAAT |
| R_18 S rRNA | CGCCGACCGAAGGGACAAGCCGA |
| F_UBQ10 | GGCCTTGTATAATCCCTGATGAAT |
| R_UBQ10 | AAAGAGATAACAGGAACGGAAACA |
